# Association between chiropractic care and use of prescription opioids among older medicare beneficiaries with spinal pain: a retrospective observational study

**DOI:** 10.1186/s12998-022-00415-7

**Published:** 2022-01-31

**Authors:** James M. Whedon, Sarah Uptmor, Andrew W. J. Toler, Serena Bezdjian, Todd A. MacKenzie, Louis A. Kazal

**Affiliations:** 1 Southern California University of Health Sciences, 16200 Amber Valley Drive, Whittier, CA 90604 USA; 2 Arizona Personal Injury Centers, Phoenix, AZ USA; 3 Geisel School of Medicine at Dartmouth, 1 Rope Ferry Rd, Hanover, NH 03755 USA

**Keywords:** Opioids, Analgesics, Chiropractic, Spinal pain, Medicare, Spinal manipulation, Aged

## Abstract

**Background:**

The burden of spinal pain can be aggravated by the hazards of opioid analgesics, which are still widely prescribed for spinal pain despite evidence-based clinical guidelines that identify non-pharmacological therapies as the preferred first-line approach. Previous studies have found that chiropractic care is associated with decreased use of opioids, but have not focused on older Medicare beneficiaries, a vulnerable population with high rates of co-morbidity and polypharmacy. The purpose of this investigation was to evaluate the association between chiropractic utilization and use of prescription opioids among older adults with spinal pain.

**Methods:**

We conducted a retrospective observational study in which we examined a nationally representative multi-year sample of Medicare claims data, 2012–2016. The study sample included 55,949 Medicare beneficiaries diagnosed with spinal pain, of whom 9,356 were recipients of chiropractic care and 46,593 were non-recipients. We measured the adjusted risk of filling a prescription for an opioid analgesic for up to 365 days following diagnosis of spinal pain. Using Cox proportional hazards modeling and inverse weighted propensity scoring to account for selection bias, we compared recipients of both primary care and chiropractic to recipients of primary care alone regarding the risk of filling a prescription.

**Results:**

The adjusted risk of filling an opioid prescription within 365 days of initial visit was 56% lower among recipients of chiropractic care as compared to non-recipients (hazard ratio 0.44; 95% confidence interval 0.40–0.49).

**Conclusions:**

Among older Medicare beneficiaries with spinal pain, use of chiropractic care is associated with significantly lower risk of filling an opioid prescription.

**Supplementary Information:**

The online version contains supplementary material available at 10.1186/s12998-022-00415-7.

## Background

The burden of spine pain among older adults in the United States (US) can be aggravated by the hazards of prescribing opioid analgesics. The most common condition for which opioids are prescribed is back pain [1]. Although spending on invasive interventions for pain in the Medicare population decreased from 2009 to 2018 [2], a parallel decrease did not occur in the use of opioid analgesics. In fact, opioid use in the aged Medicare population grew slightly from 2011 to 2016, and between 2007 and 2016, 11–14% of Medicare beneficiaries aged 65 years and older were prescribed opioids [3]. The hazards of the epidemic of opioid prescribing are particularly troublesome for older adults, who are often plagued with multiple co-morbidities and associated poly-pharmacy [4]. From 2017 to 2018, the overall rate of deaths involving any opioid decreased, but in adults over age 65, deaths due to overdose increased for both all opioids and prescription opioids [5]. Opioid analgesics continue to be widely prescribed for spinal pain despite current evidence-based clinical guidelines that identify non-pharmacological therapies as the preferred first-line approach [6].

## Chiropractic care as an alternative to opioid analgesia for spinal pain

Several non-pharmacological therapies- including spinal manipulation, therapeutic exercise, and relaxation techniques—are typically offered by chiropractors, and are recommended as first line or adjunctive therapy for spinal pain [6, 7]. The utilization of chiropractic care has been reported to be associated with reduced risk of adverse drug events in adults with spinal pain [8], and large scale observational studies have found that chiropractic care is associated with decreased use of opioids [9–11]. Among more than 101,000 adults with back pain in three contiguous New England states, the risk of filling a prescription for an opioid analgesic over a six-year period was reduced by half for recipients of chiropractic care, and the reduction in risk was greater among those who sought chiropractic care early in the course of treatment [11]. In a cohort study of more than 216,000 adults with LBP, patients who received initial treatment from chiropractors had lower odds of short-term and long-term opioid use [10]. Finally, a systematic review of six studies including a total of 62,624 subjects with spinal pain found that recipients of chiropractic care had 64% lower odds of receiving an opioid prescription as compared to non-recipients [9].

The provision of chiropractic care is inversely correlated with opioid prescriptions among younger disabled Medicare beneficiaries under 65 years of age [12]. In the study by Kazis et al. [9] 15% of subjects were beneficiaries of Medicare Advantage plans; otherwise, studies on the association between use of chiropractic care and use of opioids have been conducted on mostly younger populations of adults. Previous studies have not focused on older Medicare beneficiaries, a vulnerable and difficult to manage population with high rates of co-morbidity and polypharmacy.

## Research objective

The objective of this investigation was to evaluate the impact of chiropractic utilization upon the use of prescription opioids among Medicare beneficiaries aged 65 plus.

## Methods

We hypothesized that among older Medicare beneficiaries diagnosed with spinal pain, subjects who receive chiropractic care have a lower risk of filling a prescription for an opioid analgesic as compared to beneficiaries who do not receive chiropractic care. To test this hypothesis, we employed a retrospective cohort design to analyze Medicare administrative data collected from 2012 through 2016. Medicare is a health insurance program administered by The Centers for Medicare and Medicaid Services (CMS) of the US Department of Health and Human Services. US residents aged 65 or older are eligible for enrollment in Medicare. CMS aggregates health claims and associated administrative data and makes research datasets available to qualified research scientists. Medicare claims data include those for inpatient services (Part A), outpatient services (Part B), managed care plans (Part C), and prescription medications (Part D). The data source for this project was Medicare Parts B and D. The study was conducted according to the terms of a data use agreement between the principal investigator and CMS.

The study population was comprised of older Medicare beneficiaries, alive as of 12/31/16, living in any of the 50 US states and the District of Columbia, aged 65–99 years, continuously enrolled throughout the study period in Medicare Parts B and D. We excluded beneficiaries enrolled in Medicare Part C. The study sample was restricted to patients with office visits to a primary care physician and/or Doctor of Chiropractic for a primary diagnosis of spinal pain. A complete list of diagnosis codes used to identify and categorize spinal pain disorders may be viewed in Additional file 1: Appendix C. Diagnostic codes for non-allopathic lesions were excluded to help assure congruence between chiropractic and medical patient populations. We only included Part B claims with dates of service within calendar years 2012–2016, with payment amount greater than zero for a primary diagnosis of spine-related disorder. To enhance the validity of the recorded diagnosis, we restricted the study sample to beneficiaries with at least 2 such visits between 7 and 90 days apart. Thus, the study population included subjects with multiple office visits for spinal pain. To exclude patients with pathological pain (who would be likely to receive opioids but less likely to receive chiropractic care), we excluded beneficiaries with a primary diagnosis of cancer or receiving hospice care at any time during the study period of 2012–2016. We restricted place of service to office visits, and restricted provider specialty to family medicine, general practice, internal medicine, or chiropractic. For each subject, the first date associated with diagnosis of a spinal pain disorder was designated as the *index date*. The earliest possible index date was Jan 1, 2013. 2012 data were used to capture patient inclusion and exclusion criteria, and to calculate Charlson comorbidity scores. The latest possible index date was Dec 31, 2015, thus allowing for an observation period of 365 days for all subjects. Only the first chiropractic visit was used as a cohort inclusion criterion for Recipients; any subsequent visits did not change the subject’s primary cohort assignment or index date. We included only those subjects with Part D coverage at index date plus 365 days. We excluded all subjects with an opioid prescription fill that occurred before the index date. In our analyses of claims data, in accordance with CMS rules for analysis of health claims, cells with n < 11 were suppressed to prevent disclosure of protected health information.

Among those included in the study population we identified two cohorts of subjects: (1) Recipients of chiropractic services (*Recipients*) received both primary care and chiropractic care within 120 days of cohort inclusion. (2) *Non-recipients* received primary care but did not receive chiropractic care at any time during the study period. For the Recipients cohort, we accounted for immortal time bias by using first chiropractic visit only as a cohort inclusion criterion for Recipients; thus, subjects with an opioid prescription fill after their index date but before their first chiropractic visit were included in the Recipients cohort. We categorized spinal pain diagnoses as 1, 2, or 3 as indicators of progressively more unfavorable prognosis. [Additional file 1: Appendix C] We stratified the recipient population into three groups: (1) *Early*—patients whose first chiropractic encounter occurred within 30 days of the index date, (2) *Delayed*—first chiropractic encounter occurring within 31–90 days of the index date, and (3) *Late*—first chiropractic encounter occurring within 91–120 days of the index date. Thus, for purposes of this study, the terms *Early*, *Delayed*, and *Late* refer specifically to the timing of first chiropractic visit among Recipients of chiropractic care.

The principal outcome measure was incidence of opioid prescription fill, as recorded in Part D data. Prescription opioids were identified by the Centers for Disease Control list of opioid-containing analgesic medications and associated National Drug Codes [13]. Following aggregation of claims data and assembly of cohorts, we generated descriptive statistics by cohort on subject demographics, health status, category of spine pain diagnosis, and for Recipients, the timing of first chiropractic visit. Spine pain diagnoses were categorized as 1, 2, or 3 to broadly indicate progressively higher risk of poor outcomes.

We employed Cox proportional hazards modeling to evaluate risk of opioid prescription fill for up to 365 days following index. To assess the impact of receiving chiropractic care early in an episode of care, we sub-analyzed for risk of opioid prescription fill in the *Early*, *Delayed*, and *Late* groups of Recipients. We controlled for patient characteristics, including age, sex, race/ethnicity, state of residence, spinal pain diagnosis category, and health status at baseline as measured by Charlson comorbidity score. To reduce the effect of selection bias, we controlled for subjects’ propensity to utilize chiropractic care, using inverse probability of treatment weighting [14]. To help achieve statistical modeling that would be consistent across all measurements, both national and state-by state, propensity scores were binned into quintiles for use in the Cox proportional hazards models. The adjusted hazard ratios were estimated by including the propensity score quintiles within models as a single (categorical) variable. We did not retain data showing distribution of covariates between recipients and non-recipients before and after adjusting for propensity scores. Observational research on Medicare claims data affords a limited selection of variables for patient characteristics that can be used for propensity scoring, and as the population ages the demographics of the Medicare beneficiary population (and specifically among beneficiaries who use chiropractic care) have been slow to change.

We performed adjusted time-to-event analyses, generating hazard ratios to compare Recipients and Non-recipients regarding the risk of filling an opioid prescription. To analyze for geographic variations in outcomes, we generated hazard ratios by state. All statistical analyses were performed using SAS 9.4 (SAS Institute, Cary, NC).

## Results

**Fig. 1:**
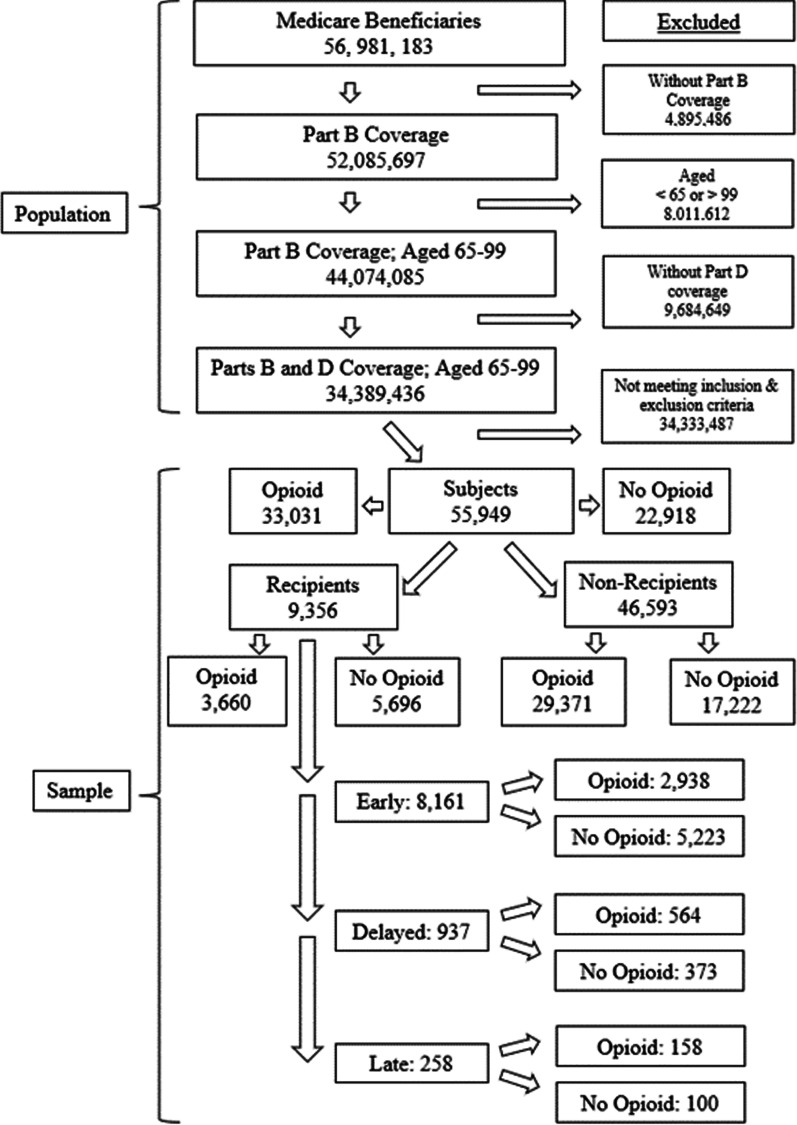
Study Population, Sampling, and Cohort Assembly. Medicare Beneficiaries = enrolled under Medicare and living thorough 2016; Part B = traditional Medicare fee-for-service outpatient coverage; Part D = Medicare pharmacy coverage; Opioid = subject with opioid prescription fill; Recipients = subject who received chiropractic care; Early = recipient of chiropractic care within 30 days following index date; Delayed = recipient of chiropractic care within 31–90 following index date; Late = recipient of chiropractic care within 91–120 following index date; Inclusion Criteria = Medicare beneficiaries alive as of 12/31/16, living in a US state or the District of Columbia, aged 65–99 years, continuously enrolled throughout the study period in Medicare Parts B, with Part D coverage at index date plus 365 days, with at least 2 office visits between 7 and 90 days apart to a primary care physician and/or Doctor of Chiropractic, claimed under Medicare Part B with date of service 2012–2016 and payment amount greater than zero for a primary diagnosis of spine-related disorder. Exclusion Criteria = beneficiaries enrolled in Medicare Part C, with primary diagnosis of cancer or receiving hospice care during 2012–2016, or opioid prescription fill that occurred before the index date

The study sample included 55,949 subjects, of whom 9356 were recipients of chiropractic care and 46,593 were non-recipients. The flow chart in Fig. 1 displays the process of sampling and cohort assembly. The cohorts differed significantly regarding all measured patient characteristics (Table 1). The majority of recipients were aged 65–74. Among subjects aged 85 and over, non-recipients greatly outnumbered recipients. Females outnumbered males by approximately 2 to 1 in both cohorts. Recipients included a higher proportion of Whites and a lower proportion of Blacks as compared to Non-recipients. Very low proportions of other racial and ethnic minorities among recipients precluded comparison with non-recipients. Regarding spinal pain diagnosis category, diagnoses in category 1 occurred with the highest frequency; category 3 diagnoses were uncommon (Table 2). Recipients included higher proportions of diagnosis category 2 cases and lower proportions of cases designated as category 1 (more favorable prognosis) and category 3 (less favorable) as compared to non-recipients. Charlson co-morbidity scores averaged significantly higher among non-recipients, indicating a greater burden of chronic disease among subjects who did not receive chiropractic care.

**Table 1:** Subject Characteristics: Medicare Part B Beneficiaries with Spinal Pain, 2012–2016

Characteristic	Non-recipients (n = 46,593)	Recipients (n = 9,356)	*P*
*Age in years: n (%)*	–	–	< 0.0001
65–74	26,189 (56.2)	6420 (68.6)	–
75–84	14,534 (31.2)	2324 (24.8)	–
85 +	5870 (12.6)	612 (6.5)	–
*Sex: n (%)*			< 0.0001
Male	14,285 (30.7)	3179 (34)	–
Female	32,308 (69.3)	6177 (66)	–
*Race/ethnicity: n (%)*	–	–	< 0.0001
Black	4722 (10.1)	342 (3.7)	–
White	34,405 (73.8)	8351 (89.3)	–
Other/ Unknown	7466 (16)	663 (7.1)	–
*Diagnosis category: n (%)*	–	–	< 0.0001
1	31,389 (67.4)	5685 (60.8)	–
2	13,975 (30)	3530 (37.7)	–
3	1229 (2.6)	141 (1.5)	
*Mean Charlson comorbidity score*	1.71	1.17	< 0.0001

Non-recipients = subjects who received Primary Care only for spinal pain; Recipients = subjects who received both Primary Care and Chiropractic Care for spinal pain; % = percentage of cohort; Charlson = Charlson Co-morbidity Score; Diagnosis Categories 1, 2, and 3 indicate progressively more unfavorable prognosis (1 = more favorable; 3 = less favorable); P = p-value

**Table 2:** Frequency of diagnoses, by category

Description	ICD-9 Code	Frequency
*Diagnostic Category 1*		
Segmental and Somatic Dysfunction of Lumbar Region	7393	1,122,329
Segmental and Somatic Dysfunction of Cervical Region	7391	862,020
Segmental and Somatic Dysfunction of Thoracic Region	7392	308,601
Segmental and Somatic Dysfunction of Sacral Region	7394	205,350
Segmental and Somatic Dysfunction of Pelvic Region	7395	105,049
Low back pain	7242	56,844
Cervicalgia	7231	42,950
Pain in Thoracic Spine	7241	15,148
Segmental and Somatic Dysfunction of Head Region	7390	17,585
Myositis	7291	6629
Disorders of Sacrum	7246	5108
*Diagnostic Category 2*		
Disc Degeneration, Lumbar	72,252	19,908
Radiculopathy	7244	18,229
Sciatica	7243	16,540
Disc Degeneration, Cervical	7224	12,967
Sprain, Lumbar	8472	9799
Radiculitis, Cervical	7234	9709
Other Intervertebral Disc Displacement, Lumbar	72,210	9434
Sprain/whiplash, Cervical	8470	8620
Cervicobrachial Syndrome	7233	8315

The table displays frequencies of the 20 most common diagnoses. Spine pain diagnoses were categorized as 1, 2, or 3 to broadly indicate progressively higher risk of poor outcomes. Category 3 diagnoses were uncommon. Only 2013 data are displayed; frequencies in other data years were similar

As shown in Table 3, the adjusted risk of filling an opioid prescription within 365 days of first office visit was 56% lower among recipients as compared to non-recipients (hazard ratio 0.44; 95% confidence interval 0.40–0.49). Throughout the study period, the results demonstrated lower risk for recipients: Hazard ratios remained within a relatively narrow range (from a high of 0.45 in 2012 to a low of 0.39 in 2015) with similarly narrow confidence intervals [Additional file 1: Appendix A]. Analysis of hazard ratios by state failed to generate statistically significant results in all but 16 states and revealed no strong discernible national pattern of geographic variation. However, many of the higher hazard ratios, indicating less reduction in risk, are observable in Southern states where chiropractic supply and utilization are relatively low [15, 16], and a block of states with greater risk reduction, demonstrated by very low hazard ratios, is seen in northern prairie states where chiropractic supply and utilization are relatively high [Additional file 1: Appendix B].

**Table 3:** Adjusted risk of opioid prescription fill among recipients and subgroups vs. non-recipients

Cohort: n (%)	Fill: n (%)	No Fill: n (%)	Hazard Ratio	95% CI
Total	33,031 (59)	22,918 (41)	–	–
Non-recipients 46,593 (83)	29,371 (52)	17,222 (31)	(referent)	–
Recipients 9356 (17)	3660 (7)	5696 (10)		
*Unadjusted model*			0.46	0.44–0.47
*Adjusted for state; without propensity score*			0.49	0.47–0.50
*Full model: with propensity score, without state*			0.39	0.36–0.43
*Full model with both state and propensity score*			0.44	0.40–0.49
Recipients, Early 8,161 (15)	2,938 (5)	5,223 (9)	0.38	0.34–0.42
Recipients, Delayed 937 (2)	564 (1)	373 (1)	0.90	0.80–1.03
Recipients, Late 258 (< 1)	158 (< 1)	100 (< 1)	0.93	0.77–1.13

Results of Cox proportional hazards modeling for isk of opioid prescription fill for up to 365 days following index. Full model controlled for for patient age, sex, race/ethnicity, state of residence, spinal pain diagnosis category, and Charlson comorbidity score. Non-recipients = subjects who received Primary Care only for spinal pain; Recipients = subjects who received both Primary Care and Chiropractic Care for spinal pain; % = percentage of total study sample; HR = Hazard Ratio; The hazard ratios quantify risk of receiving a prescription opioid within 365 days of initial visit. A hazard ratio of 1.0 signifies equal risk between groups: as the number decreases from 1.0 it signifies decreased risk of filling an opioid prescription. LCL = lower confidence limit; UCL = upper confidence limit; Early = subjects who received chiropractic care within 30 days following diagnosis of spinal disorder; Delayed = subjects who received chiropractic care at 31–90 days following diagnosis of spinal disorder; Late = subjects who received chiropractic care at 91–120 days following diagnosis of spinal disorder

**Fig. 2:**
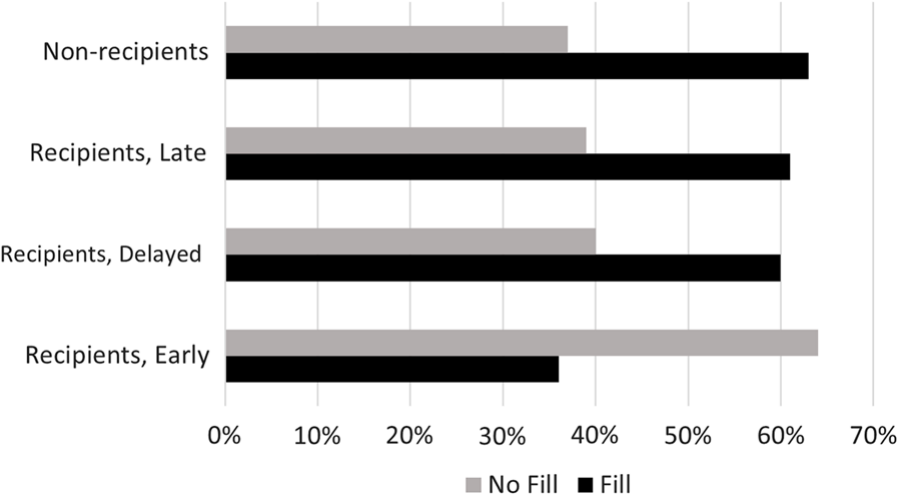
Percentage of Subjects with Opioid Prescription Fill vs. No Fill by Cohort and Sub-group. Fill = prescription fill for opioid analgesic medication; Recipients = subjects who received both primary care and chiropractic care; Non-Recipients = subjects who received primary care and no chiropractic care; Recipients, Early = subjects who received chiropractic care within 30 days following diagnosis of spinal disorder; Recipients, Delayed = subjects who received chiropractic care at 31–90 days following diagnosis of spinal disorder; Recipients, Late = subjects who received chiropractic care at 91–120 days following diagnosis of spinal disorder

The lower risk was not consistent across all subgroups of recipients. Figure 2 compares non-recipients to subgroups of recipients for rates of filling opioid prescriptions. The greatest proportion of those who avoided filling an opioid prescription was seen in the Early sub-group, which comprised 87% of all recipients of chiropractic care. Among early recipients of chiropractic care, the adjusted risk of filling an opioid prescription was 62% lower as compared to non-recipients (hazard ratio 0.38; 95% confidence interval 0.34- 0.42). In the Delayed and Late sub-groups, we found no statistically significant difference in fill rates as compared to non-recipients.

**Fig. 3:**
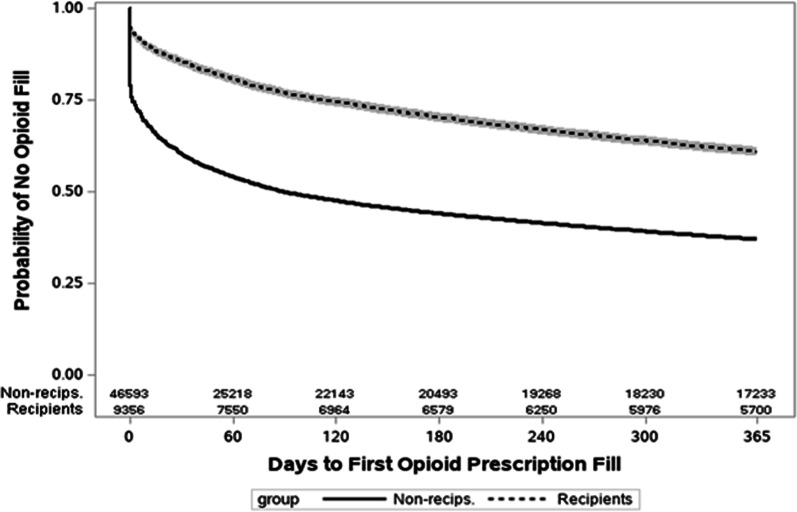
One-year Adjusted Risk of Opioid Prescription Fill among Recipients of Chiropractic Care as Compared to Non-recipients, with Number of Subjects at Risk and 95% Hall-Wellner Bands. The figure illustrates adjusted hazard ratios in a time-to-event analysis. Opioid Fill = Prescription fill for opioid analgesic medication; Recipients = subjects who received both primary care and chiropractic care; Non-Recips. = Non-Recipients (subjects who received primary care and no chiropractic care); Risk is expressed as direct adjusted survivor functions with 95% confidence limits

Figure 3 depicts the comparative association between choice of treatment and risk of opioid use via survival curves, in which “survival” is equivalent to going without filling a prescription for an opioid analgesic. Overall, for the entire study period, recipients maintained a consistent advantage over non-recipients at up to 365 days following diagnosis of spinal pain.

## Discussion

The results of this study support our hypothesis that among older Medicare beneficiaries diagnosed with spinal pain, recipients of chiropractic care have a lower risk of filling a prescription for an opioid analgesic as compared to non-recipients. The findings are generalizable to older US adults. Based upon the results in Table 3, calculation of number needed to treat shows that, on average, 2.2 patients would have to switch from primary medical care alone to primary care plus chiropractic care for one additional patient to not fill a prescription for opioid medication. Lower risk of filling an opioid prescription may have been driven by the Early sub-group of recipients, who initiated chiropractic care within the first 30 days of an episode of spinal pain. These results are consistent with previous studies on other, mostly younger populations, who received early chiropractic care, physical therapy, or other non-pharmacological pain management [9–11, 17, 18].

The observed advantage of early chiropractic care mirrors the results of a prior study on a population of adults aged 18–84 [11]. Similarly, a systematic review by Corcoran et al. reported 64% lower odds of opioid prescription among recipients, for whom a chiropractor was either the first provider seen or part of the initial treatment strategy in four of the six included studies [9]. Previous studies have reported other benefits associated with seeing a chiropractor first. Keeney et al. reported that among patients with work-related back injuries, more than 42% of workers who first saw a surgeon underwent surgery for their injury, as compared to less than 2% of those who first saw a chiropractor [19]. In a retrospective analysis of health claims for patients with low back pain, Liliedahl et al. found that total insurance payments for episodes of care initiated with a chiropractor were almost 40% lower than episodes initiated with a medical doctor, and remained 20% less expensive following risk adjustment [20]. Thus, our results suggest that—in addition to lower cost and more efficient utilization of clinical resources—early chiropractic care for spinal pain is also associated with improved patient safety as compared to conventional medical care, at least with regard to use of opioids.

Because measures of opioid prescribing have been reported to vary two- to three-fold by state [21], we controlled for beneficiary state of residence. Putting geography on a statistically level ground prevents masking of the effects of confounders. However, at the national level, the effect of chiropractic care on use of prescription opioids appears to be such a strong relationship that associations with geography are barely noticeable.

There is a critical need for high value non-pharmacological approaches to pain management [22]. Cost comparisons between chiropractic care and opioid analgesic therapy for spinal problems favor the value of the former [23, 24], with achievement of similar if not better clinical outcomes [25]. To increase the use of high value treatments and reduce the utilization of low value spine care, clinicians and policy makers should align decisions with current guidelines [26]. The results of this study confirm previous reports that evidence-based non-pharmacological approaches to spine care appear to offer not only alternatives to prescription opioids, but upstream strategies for curtailing the use and misuse of opioid medications [27, 28].

## Limitations

Because Medicare Part C claims data are not readily available for research, they were not included in our analyses. This exclusion may have introduced bias due to differences in subject characteristics between the two groups: Part C beneficiaries tend to be more prosperous because Part C premiums are higher. However, Part B beneficiaries greatly outnumber those in Part C. Spinal manipulation is the only type of treatment that is currently reimbursable to chiropractors under Medicare Part B [29]. Thus, under Medicare chiropractic is equated with spinal manipulation. It is possible that the use of opioids may have been confounded by indication (i.e., prescription of opioids for a condition other than spine pain). We endeavored to limit such confounding by excluding cancer and hospice patients. Also, unmeasured changes in patient choice of treatment could have accounted for changes in the likelihood of filling an opioid prescription. Insufficient adjustment of confounding factors may have occurred due to limited availability of variables in the existing datasets. However, the rigorous methods employed—particularly the one-year risk estimates generated by adjusted time-to-event analyses—allow stronger inference of causality as compared to simple correlative studies.

Because chiropractors commonly treat pain conditions other than spine pain, chiropractic care may be expected to impact use of analgesics for both spinal and extraspinal conditions. We reduced the effect of selection bias through use of propensity scoring, weighted by inverse probability of treatment; the weighted approach helps maintain sample size and preserve external validity [14]. Finally, it should be noted that this study drew from data on opioids prescribed through 2016. As the opioids crisis has evolved, and subsequent to the publication in 2016 of the Centers for Disease Control (CDC) guidelines on prescribing opioids [30], many physicians have adopted more conservative prescribing practices, and the hazards once ascribed to prescription analgesic medications have shifted toward drugs such as Fentanyl that are often obtained illegally without prescription [31]. In response to this trend, the American Medical Association (AMA) urged the CDC in 2020 to revise its prescribing guidelines and remove arbitrary limits and restrictions on opioid prescribing [32], but in 2021 the professional organization, *Physicians for Responsible Opioid Prescribing* rejected the AMA’s claim that the US no longer has a prescription opioid driven epidemic [33]. The CDC studied more than 180 million emergency department visits from Dec. 2018, to Oct. 2020, and found that opioid overdoses increased by 29% during the COVID-19 pandemic [31]. Thus, the need for upstream alternatives to opioid prescribing remains critical, and the preponderance of evidence in favor of chiropractic points to investigations on systematic dissemination and implementation as the next step in research.

## Conclusions

Older Medicare enrollees with spinal pain who saw both a chiropractor and a primary care physician had less than half the risk of filling an opioid prescription, as compared to those who received primary medical care alone. The association was most pronounced among those who saw a chiropractor within the first 30 days of care.

## Supplementary Information

**Additional file 1: APPENDIX A.** Risk of Opioid Prescription Fill Among Recipients vs. Non-recipients, by Year. **APPENDIX B.** Geographic Variation in Risk of Opioid Prescription Fill: Recipients vs. Non-recipients. **APPENDIX C.** Categorized Diagnosis Codes.

## Data Availability

The data that support the findings of this study were obtained from The Centers for Medicare and Medicaid Services (CMS), U.S. Department of Health and Human Services. These data were accessed by the principal investigator under the terms of a data use agreement with CMS, which strictly prohibits sharing of data; thus, the data are not publicly available.

## Abbreviations

CMS: Centers for Medicare and Medicaid Services
CDC: Centers for Disease Control
AMA: American Medical Association
