# Supplementary material for: Association between chiropractic care and use of prescription opioids among older medicare beneficiaries with spinal pain: a retrospective observational study
Source: Chiropr Man Therap. 2022 Jan 31;30:5. doi: 10.1186/s12998-022-00415-7 (PMC8802278; doi:10.1186/s12998-022-00415-7)
Supplement: Supplementary file 1 — Additional file 1: APPENDIX A. Risk of Opioid Prescription Fill Among Recipients vs. Non-recipients, by Year. APPENDIX B. Geographic Variation in Risk of Opioid Prescription Fill: Recipients vs. Non-recipients. APPENDIX C. Categorized Diagnosis Codes. [file 12998_2022_415_MOESM1_ESM.docx]

**APPENDIX A. Risk of Opioid Prescription Fill Among Recipients vs. Non-recipients, by Year***
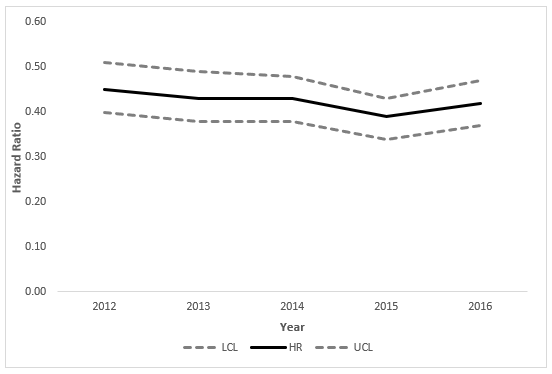
*

*Recipients = subjects who received both primary care and chiropractic care; Non-Recipients = subjects who received primary care and no chiropractic care); LCL = lower confidence limit; HR = hazard ratio; UCL = upper confidence limit*

**APPENDIX B. Geographic Variation in Risk of Opioid Prescription Fill: Recipients vs. Non-recipients

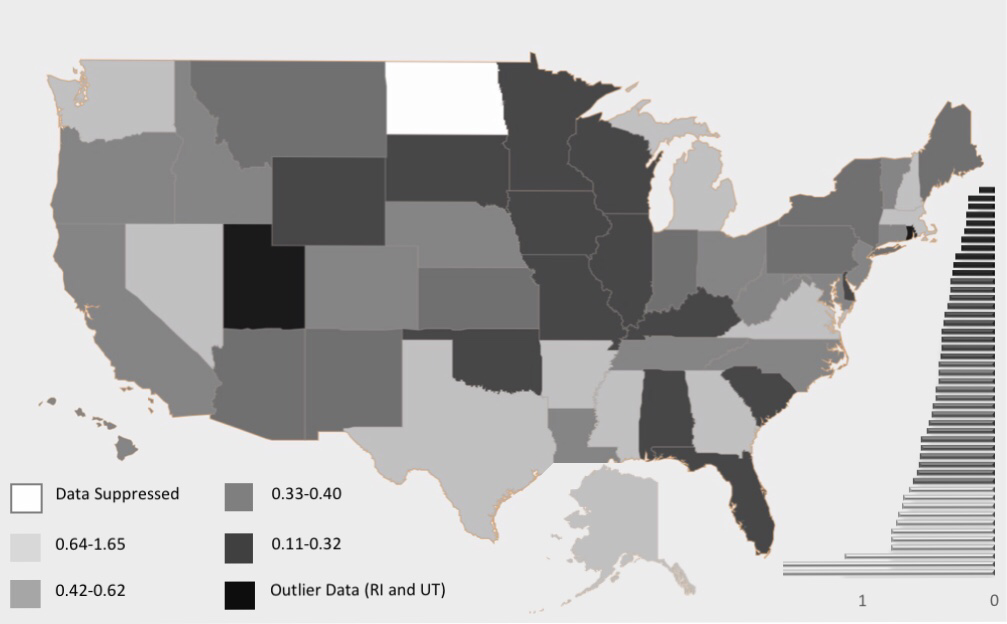
**

*The hazard ratios quantify risk of receiving a prescription opioid within 365 days of initial visit. A hazard ratio of 1.0 would mean the two groups have equal risk, as the number decreases from 1.0 it shows a decreased risk of filling an opioid prescription. The states are shaded in gray scale by quartile of hazard ratio (lightest shade = lowest quartile (least risk reduction); darkest shade = highest quartile (greatest risk reduction). The bar graph shows the relative ranges of each quartile (gray-scale shading correlates with quartiles in the legend). Outlier hazard ratios for Utah and Rhode Island are not represented in the bar graph. Data for North Dakota was suppressed in accordance with CMS requirements. Hawaii and Alaska not to scale.*

**APPENDIX C.**

**Categorized Diagnosis Codes**

|  |  |  |  |  |
| --- | --- | --- | --- | --- |
| **Diagnosis Category** | **ICD-9 Code** | **ICD-9 Brief Description** | **ICD-10 Code** | **ICD-10 Brief Description** |
| 1 | 721 | Spondylosis | M47.812 | Spondylosis without myelopathy or radiculopathy, cervical region |
| 1 | 721.2 | Spondylosis | M47.814 | Spondylosis without myelopathy or radiculopathy, thoracic region |
| 1 | 721.3 | Spondylosis | M47.817 | Spondylosis without myelopathy or radiculopathy, lumbosacral region |
| 1 | 723.1 | Pain, Neck | M54.2 | Cervicalgia |
| 1 | 724.1 | Pain Thoracic Spine | M54.6 | Pain in thoracic spine |
| 1 | 724.2 | Pain lower back | M54.5 | Low back pain |
| 1 | 724.6 | Disorders of Sacrum | M53.3 | Sacrococcygeal disorders, not elsewhere classified |
| 1 | 724.79 | Coccyxgodynia | M53.3 | Sacrococcygeal disorders, not elsewhere classified |
| 1 | 729.1 | Myositis | M609 | Myositis, unspecified |
| 1 | 729.1 | Myositis | M791 | Myalgia |
| 1 | 729.1 | Myositis | M797 | Fibromyalgia |
| 1 | 733.6 | Costochondritis | M94.0 | Chondrocostal junction syndrome [Tietze] |
| 1 | 737.1 | Kyphosis | M40.00 | Postural kyphosis, site unspecified |
| 1 | 737.1 | Kyphosis | M40.209 | Unspecified kyphosis, site unspecified |
| 1 | 737.3 | Scoliosis w/o neurogenic cause | M41.20 | Other idiopathic scoliosis, site unspecified |
| 1 | 739.0 | Nonallopathic Lesions of Head Region | M9900 | Segmental and Somatic Dysfunction of Head Region |
| 1 | 739.1 | Nonallopathic Lesions of Cervical Region | M9901 | Segmental and Somatic Dysfunction of Cervical Region |
| 1 | 739.2 | Nonallopathic Lesions of Thoracic Region | M9902 | Segmental and Somatic Dysfunction of Thoracic Region |
| 1 | 739.3 | Nonallopathic Lesions of Lumbar Region | M9903 | Segmental and Somatic Dysfunction of Lumbar Region |
| 1 | 739.4 | Nonallopathic Lesions of Sacral Region | M9904 | Segmental and Somatic Dysfunction of Sacral Region |
| 1 | 739.5 | Nonallopathic Lesions of Pelvic Region | M9905 | Segmental and Somatic Dysfunction of Pelvic Region |
| 1 | 847.3 | Strain, non traumatic musculoskeletal | S33.800A | Sprain of other parts of lumbar spine and pelvis, initial encounter |
| 1 | 847.3 | Strain, non traumatic musculoskeletal | S33.801A | Sprain of other parts of lumbar spine and pelvis, initial encounter |
| 1 | 847.3 | Strain, non traumatic musculoskeletal | S33.802A | Sprain of other parts of lumbar spine and pelvis, initial encounter |
| 1 | 847.3 | Strain, non traumatic musculoskeletal | S33.803A | Sprain of other parts of lumbar spine and pelvis, initial encounter |
| 1 | 847.3 | Strain, non traumatic musculoskeletal | S33.804A | Sprain of other parts of lumbar spine and pelvis, initial encounter |
| 1 | 847.3 | Strain, non traumatic musculoskeletal | S33.805A | Sprain of other parts of lumbar spine and pelvis, initial encounter |
| 1 | 847.3 | Strain, non traumatic musculoskeletal | S33.806A | Sprain of other parts of lumbar spine and pelvis, initial encounter |
| 1 | 847.3 | Strain, non traumatic musculoskeletal | S33.807A | Sprain of other parts of lumbar spine and pelvis, initial encounter |
| 1 | 847.3 | Strain, non traumatic musculoskeletal | S33.808A | Sprain of other parts of lumbar spine and pelvis, initial encounter |
| 1 | 847.3 | Strain, non traumatic musculoskeletal | S33.809A | Sprain of other parts of lumbar spine and pelvis, initial encounter |
| 1 | 847.3 | Strain, non traumatic musculoskeletal | S33.810A | Sprain of other parts of lumbar spine and pelvis, initial encounter |
| 1 | 847.3 | Strain, non traumatic musculoskeletal | S33.811A | Sprain of other parts of lumbar spine and pelvis, initial encounter |
| 1 | 847.3 | Strain, non traumatic musculoskeletal | S33.812A | Sprain of other parts of lumbar spine and pelvis, initial encounter |
| 1 | 847.3 | Strain, non traumatic musculoskeletal | S33.813A | Sprain of other parts of lumbar spine and pelvis, initial encounter |
| 1 | 847.3 | Strain, non traumatic musculoskeletal | S33.814A | Sprain of other parts of lumbar spine and pelvis, initial encounter |
| 1 | 847.3 | Strain, non traumatic musculoskeletal | S33.815A | Sprain of other parts of lumbar spine and pelvis, initial encounter |
| 1 | 847.3 | Strain, non traumatic musculoskeletal | S33.816A | Sprain of other parts of lumbar spine and pelvis, initial encounter |
| 1 | 847.3 | Strain, non traumatic musculoskeletal | S33.817A | Sprain of other parts of lumbar spine and pelvis, initial encounter |
| 1 | 847.3 | Strain, non traumatic musculoskeletal | S33.818A | Sprain of other parts of lumbar spine and pelvis, initial encounter |
| 1 | 847.3 | Strain, non traumatic musculoskeletal | S33.819A | Sprain of other parts of lumbar spine and pelvis, initial encounter |
| 1 | 847.3 | Strain, non traumatic musculoskeletal | S33.820A | Sprain of other parts of lumbar spine and pelvis, initial encounter |
| 1 | 847.3 | Strain, non traumatic musculoskeletal | S33.821A | Sprain of other parts of lumbar spine and pelvis, initial encounter |
| 1 | 847.3 | Strain, non traumatic musculoskeletal | S33.822A | Sprain of other parts of lumbar spine and pelvis, initial encounter |
| 1 | 847.3 | Strain, non traumatic musculoskeletal | S33.823A | Sprain of other parts of lumbar spine and pelvis, initial encounter |
| 1 | 847.3 | Strain, non traumatic musculoskeletal | S33.824A | Sprain of other parts of lumbar spine and pelvis, initial encounter |
| 1 | 847.3 | Strain, non traumatic musculoskeletal | S33.825A | Sprain of other parts of lumbar spine and pelvis, initial encounter |
| 1 | 847.3 | Strain, non traumatic musculoskeletal | S33.826A | Sprain of other parts of lumbar spine and pelvis, initial encounter |
| 1 | 847.3 | Strain, non traumatic musculoskeletal | S33.827A | Sprain of other parts of lumbar spine and pelvis, initial encounter |
| 1 | 847.3 | Strain, non traumatic musculoskeletal | S33.828A | Sprain of other parts of lumbar spine and pelvis, initial encounter |
| 1 | 847.3 | Strain, non traumatic musculoskeletal | S33.829A | Sprain of other parts of lumbar spine and pelvis, initial encounter |
| 1 | 847.3 | Strain, non traumatic musculoskeletal | S33.830A | Sprain of other parts of lumbar spine and pelvis, initial encounter |
| 1 | 847.3 | Strain, non traumatic musculoskeletal | S33.831A | Sprain of other parts of lumbar spine and pelvis, initial encounter |
| 1 | 847.3 | Strain, non traumatic musculoskeletal | S33.832A | Sprain of other parts of lumbar spine and pelvis, initial encounter |
| 1 | 847.3 | Strain, non traumatic musculoskeletal | S33.833A | Sprain of other parts of lumbar spine and pelvis, initial encounter |
| 1 | 847.3 | Strain, non traumatic musculoskeletal | S33.834A | Sprain of other parts of lumbar spine and pelvis, initial encounter |
| 1 | 847.3 | Strain, non traumatic musculoskeletal | S33.835A | Sprain of other parts of lumbar spine and pelvis, initial encounter |
| 1 | 847.3 | Strain, non traumatic musculoskeletal | S33.836A | Sprain of other parts of lumbar spine and pelvis, initial encounter |
| 1 | 847.3 | Strain, non traumatic musculoskeletal | S33.837A | Sprain of other parts of lumbar spine and pelvis, initial encounter |
| 1 | 847.3 | Strain, non traumatic musculoskeletal | S33.838A | Sprain of other parts of lumbar spine and pelvis, initial encounter |
| 1 | 847.3 | Strain, non traumatic musculoskeletal | S33.839A | Sprain of other parts of lumbar spine and pelvis, initial encounter |
| 1 | 847.3 | Strain, non traumatic musculoskeletal | S33.840A | Sprain of other parts of lumbar spine and pelvis, initial encounter |
| 1 | 847.3 | Strain, non traumatic musculoskeletal | S33.841A | Sprain of other parts of lumbar spine and pelvis, initial encounter |
| 1 | 847.3 | Strain, non traumatic musculoskeletal | S33.842A | Sprain of other parts of lumbar spine and pelvis, initial encounter |
| 1 | 847.3 | Strain, non traumatic musculoskeletal | S33.843A | Sprain of other parts of lumbar spine and pelvis, initial encounter |
| 1 | 847.3 | Strain, non traumatic musculoskeletal | S33.844A | Sprain of other parts of lumbar spine and pelvis, initial encounter |
| 1 | 847.3 | Strain, non traumatic musculoskeletal | S33.845A | Sprain of other parts of lumbar spine and pelvis, initial encounter |
| 1 | 847.3 | Strain, non traumatic musculoskeletal | S33.846A | Sprain of other parts of lumbar spine and pelvis, initial encounter |
| 1 | 847.3 | Strain, non traumatic musculoskeletal | S33.847A | Sprain of other parts of lumbar spine and pelvis, initial encounter |
| 1 | 847.3 | Strain, non traumatic musculoskeletal | S33.848A | Sprain of other parts of lumbar spine and pelvis, initial encounter |
| 1 | 847.3 | Strain, non traumatic musculoskeletal | S33.849A | Sprain of other parts of lumbar spine and pelvis, initial encounter |
| 1 | 847.3 | Strain, non traumatic musculoskeletal | S33.850A | Sprain of other parts of lumbar spine and pelvis, initial encounter |
| 1 | 847.3 | Strain, non traumatic musculoskeletal | S33.851A | Sprain of other parts of lumbar spine and pelvis, initial encounter |
| 1 | 847.3 | Strain, non traumatic musculoskeletal | S33.852A | Sprain of other parts of lumbar spine and pelvis, initial encounter |
| 1 | 847.3 | Strain, non traumatic musculoskeletal | S33.853A | Sprain of other parts of lumbar spine and pelvis, initial encounter |
| 1 | 847.3 | Strain, non traumatic musculoskeletal | S33.854A | Sprain of other parts of lumbar spine and pelvis, initial encounter |
| 1 | 847.3 | Strain, non traumatic musculoskeletal | S33.855A | Sprain of other parts of lumbar spine and pelvis, initial encounter |
| 1 | 847.3 | Strain, non traumatic musculoskeletal | S33.856A | Sprain of other parts of lumbar spine and pelvis, initial encounter |
| 1 | 847.3 | Strain, non traumatic musculoskeletal | S33.857A | Sprain of other parts of lumbar spine and pelvis, initial encounter |
| 1 | 847.3 | Strain, non traumatic musculoskeletal | S33.858A | Sprain of other parts of lumbar spine and pelvis, initial encounter |
| 1 | 847.3 | Strain, non traumatic musculoskeletal | S33.859A | Sprain of other parts of lumbar spine and pelvis, initial encounter |
| 1 | 847.3 | Strain, non traumatic musculoskeletal | S33.860A | Sprain of other parts of lumbar spine and pelvis, initial encounter |
| 1 | 847.3 | Strain, non traumatic musculoskeletal | S33.861A | Sprain of other parts of lumbar spine and pelvis, initial encounter |
| 1 | 847.3 | Strain, non traumatic musculoskeletal | S33.862A | Sprain of other parts of lumbar spine and pelvis, initial encounter |
| 1 | 847.3 | Strain, non traumatic musculoskeletal | S33.863A | Sprain of other parts of lumbar spine and pelvis, initial encounter |
| 1 | 847.3 | Strain, non traumatic musculoskeletal | S33.864A | Sprain of other parts of lumbar spine and pelvis, initial encounter |
| 1 | 847.3 | Strain, non traumatic musculoskeletal | S33.865A | Sprain of other parts of lumbar spine and pelvis, initial encounter |
| 1 | 847.3 | Strain, non traumatic musculoskeletal | S33.866A | Sprain of other parts of lumbar spine and pelvis, initial encounter |
| 1 | 847.3 | Strain, non traumatic musculoskeletal | S33.867A | Sprain of other parts of lumbar spine and pelvis, initial encounter |
| 1 | 847.3 | Strain, non traumatic musculoskeletal | S33.868A | Sprain of other parts of lumbar spine and pelvis, initial encounter |
| 1 | 847.3 | Strain, non traumatic musculoskeletal | S33.869A | Sprain of other parts of lumbar spine and pelvis, initial encounter |
| 1 | 847.3 | Strain, non traumatic musculoskeletal | S33.870A | Sprain of other parts of lumbar spine and pelvis, initial encounter |
| 1 | 847.3 | Strain, non traumatic musculoskeletal | S33.871A | Sprain of other parts of lumbar spine and pelvis, initial encounter |
| 1 | 847.3 | Strain, non traumatic musculoskeletal | S33.872A | Sprain of other parts of lumbar spine and pelvis, initial encounter |
| 1 | 847.3 | Strain, non traumatic musculoskeletal | S33.873A | Sprain of other parts of lumbar spine and pelvis, initial encounter |
| 1 | 847.3 | Strain, non traumatic musculoskeletal | S33.874A | Sprain of other parts of lumbar spine and pelvis, initial encounter |
| 1 | 847.3 | Strain, non traumatic musculoskeletal | S33.875A | Sprain of other parts of lumbar spine and pelvis, initial encounter |
| 1 | 847.3 | Strain, non traumatic musculoskeletal | S33.876A | Sprain of other parts of lumbar spine and pelvis, initial encounter |
| 1 | 847.3 | Strain, non traumatic musculoskeletal | S33.877A | Sprain of other parts of lumbar spine and pelvis, initial encounter |
| 1 | 847.3 | Strain, non traumatic musculoskeletal | S33.878A | Sprain of other parts of lumbar spine and pelvis, initial encounter |
| 1 | 847.3 | Strain, non traumatic musculoskeletal | S33.879A | Sprain of other parts of lumbar spine and pelvis, initial encounter |
| 1 | 847.3 | Strain, non traumatic musculoskeletal | S33.880A | Sprain of other parts of lumbar spine and pelvis, initial encounter |
| 1 | 847.3 | Strain, non traumatic musculoskeletal | S33.881A | Sprain of other parts of lumbar spine and pelvis, initial encounter |
| 1 | 847.3 | Strain, non traumatic musculoskeletal | S33.882A | Sprain of other parts of lumbar spine and pelvis, initial encounter |
| 1 | 847.3 | Strain, non traumatic musculoskeletal | S33.883A | Sprain of other parts of lumbar spine and pelvis, initial encounter |
| 1 | 847.3 | Strain, non traumatic musculoskeletal | S33.884A | Sprain of other parts of lumbar spine and pelvis, initial encounter |
| 1 | 847.3 | Strain, non traumatic musculoskeletal | S33.885A | Sprain of other parts of lumbar spine and pelvis, initial encounter |
| 1 | 847.3 | Strain, non traumatic musculoskeletal | S33.886A | Sprain of other parts of lumbar spine and pelvis, initial encounter |
| 1 | 847.3 | Strain, non traumatic musculoskeletal | S33.887A | Sprain of other parts of lumbar spine and pelvis, initial encounter |
| 1 | 847.3 | Strain, non traumatic musculoskeletal | S33.888A | Sprain of other parts of lumbar spine and pelvis, initial encounter |
| 1 | 847.3 | Strain, non traumatic musculoskeletal | S33.889A | Sprain of other parts of lumbar spine and pelvis, initial encounter |
| 1 | 847.3 | Strain, non traumatic musculoskeletal | S33.890A | Sprain of other parts of lumbar spine and pelvis, initial encounter |
| 1 | 847.3 | Strain, non traumatic musculoskeletal | S33.891A | Sprain of other parts of lumbar spine and pelvis, initial encounter |
| 1 | 847.3 | Strain, non traumatic musculoskeletal | S33.892A | Sprain of other parts of lumbar spine and pelvis, initial encounter |
| 1 | 847.3 | Strain, non traumatic musculoskeletal | S33.893A | Sprain of other parts of lumbar spine and pelvis, initial encounter |
| 1 | 847.3 | Strain, non traumatic musculoskeletal | S33.894A | Sprain of other parts of lumbar spine and pelvis, initial encounter |
| 1 | 847.3 | Strain, non traumatic musculoskeletal | S33.895A | Sprain of other parts of lumbar spine and pelvis, initial encounter |
| 1 | 847.3 | Strain, non traumatic musculoskeletal | S33.896A | Sprain of other parts of lumbar spine and pelvis, initial encounter |
| 1 | 847.3 | Strain, non traumatic musculoskeletal | S33.897A | Sprain of other parts of lumbar spine and pelvis, initial encounter |
| 1 | 847.3 | Strain, non traumatic musculoskeletal | S33.898A | Sprain of other parts of lumbar spine and pelvis, initial encounter |
| 1 | 847.3 | Strain, non traumatic musculoskeletal | S33.899A | Sprain of other parts of lumbar spine and pelvis, initial encounter |
| 2 | 722 | Disc herniation | M50.20 | Other cervical disc displacement, unspecified cervical region |
| 2 | 722.1 | Disc herniation | M51.26 | Other intervertebral disc displacement, lumbar region |
| 2 | 722.1 | Disc herniation | M51.27 | Other intervertebral disc displacement, lumbosacral region |
| 2 | 722.11 | Disc herniation | M51.24 | Other intervertebral disc displacement, thoracic region |
| 2 | 722.11 | Disc herniation | M51.25 | Other intervertebral disc displacement, thoracolumbar region |
| 2 | 722.4 | Disc degeneration | M50.30 | Other cervical disc degeneration, unspecified cervical region |
| 2 | 722.51 | Disc degeneration | M51.34 | Other intervertebral disc degeneration, thoracic region |
| 2 | 722.51 | Disc degeneration | M51.35 | Other intervertebral disc degeneration, thoracolumbar region |
| 2 | 722.52 | Disc degeneration | M51.36 | Other intervertebral disc degeneration, lumbar region |
| 2 | 722.52 | Disc degeneration | M51.37 | Other intervertebral disc degeneration, lumbosacral region |
| 2 | 723 | Spinal stenosis | M48.02 | Spinal stenosis, cervical region |
| 2 | 723.3 | Cervicobrachial Syndrome | M531 | Cervicobrachial Syndrome |
| 2 | 723.4 | Radiculitis | M54.12 | Radiculopathy, cervical region |
| 2 | 723.4 | Radiculitis | M54.13 | Radiculopathy, cervicothoracic region |
| 2 | 724.01 | Spinal stenosis | M48.04 | Spinal stenosis, thoracic region |
| 2 | 724.02 | Spinal stenosis | M48.06 | Spinal stenosis, lumbar region |
| 2 | 724.3 | Sciatica | M54.30 | Sciatica, unspecified site |
| 2 | 724.4 | Pain, Radicular | M54.14 | Radiculopathy, thoracic region |
| 2 | 724.4 | Pain, Radicular | M54.15 | Radiculopathy, thoracolumbar region |
| 2 | 724.4 | Radiculitis | M54.14 | Radiculopathy, thoracic region |
| 2 | 724.4 | Pain, Radicular | M54.16 | Radiculopathy, lumbar region |
| 2 | 724.4 | Pain, Radicular | M54.17 | Radiculopathy, lumbosacral region |
| 2 | 724.4 | Radiculitis | M54.15 | Radiculopathy, thoracolumbar region |
| 2 | 724.4 | Radiculitis | M54.16 | Radiculopathy, lumbar region |
| 2 | 724.4 | Radiculitis | M54.17 | Radiculopathy, lumbosacral region |
| 2 | 738.4 | Spondylolisthesis | M43.10 | Spondylolisthesis, site unspecified |
| 2 | 756.12 | Spondylolisthesis congenital | Q76.2 | Congenital spondylolisthesis |
| 2 | 847 | Sprain/whiplash | S13.400A | Sprain of ligaments of cervical spine, initial encounter |
| 2 | 847 | Sprain/whiplash | S13.401A | Sprain of ligaments of cervical spine, initial encounter |
| 2 | 847 | Sprain/whiplash | S13.402A | Sprain of ligaments of cervical spine, initial encounter |
| 2 | 847 | Sprain/whiplash | S13.403A | Sprain of ligaments of cervical spine, initial encounter |
| 2 | 847 | Sprain/whiplash | S13.404A | Sprain of ligaments of cervical spine, initial encounter |
| 2 | 847 | Sprain/whiplash | S13.405A | Sprain of ligaments of cervical spine, initial encounter |
| 2 | 847 | Sprain/whiplash | S13.406A | Sprain of ligaments of cervical spine, initial encounter |
| 2 | 847 | Sprain/whiplash | S13.407A | Sprain of ligaments of cervical spine, initial encounter |
| 2 | 847 | Sprain/whiplash | S13.408A | Sprain of ligaments of cervical spine, initial encounter |
| 2 | 847 | Sprain/whiplash | S13.409A | Sprain of ligaments of cervical spine, initial encounter |
| 2 | 847 | Sprain/whiplash | S13.410A | Sprain of ligaments of cervical spine, initial encounter |
| 2 | 847 | Sprain/whiplash | S13.411A | Sprain of ligaments of cervical spine, initial encounter |
| 2 | 847 | Sprain/whiplash | S13.412A | Sprain of ligaments of cervical spine, initial encounter |
| 2 | 847 | Sprain/whiplash | S13.413A | Sprain of ligaments of cervical spine, initial encounter |
| 2 | 847 | Sprain/whiplash | S13.414A | Sprain of ligaments of cervical spine, initial encounter |
| 2 | 847 | Sprain/whiplash | S13.415A | Sprain of ligaments of cervical spine, initial encounter |
| 2 | 847 | Sprain/whiplash | S13.416A | Sprain of ligaments of cervical spine, initial encounter |
| 2 | 847 | Sprain/whiplash | S13.417A | Sprain of ligaments of cervical spine, initial encounter |
| 2 | 847 | Sprain/whiplash | S13.418A | Sprain of ligaments of cervical spine, initial encounter |
| 2 | 847 | Sprain/whiplash | S13.419A | Sprain of ligaments of cervical spine, initial encounter |
| 2 | 847 | Sprain/whiplash | S13.420A | Sprain of ligaments of cervical spine, initial encounter |
| 2 | 847 | Sprain/whiplash | S13.421A | Sprain of ligaments of cervical spine, initial encounter |
| 2 | 847 | Sprain/whiplash | S13.422A | Sprain of ligaments of cervical spine, initial encounter |
| 2 | 847 | Sprain/whiplash | S13.423A | Sprain of ligaments of cervical spine, initial encounter |
| 2 | 847 | Sprain/whiplash | S13.424A | Sprain of ligaments of cervical spine, initial encounter |
| 2 | 847 | Sprain/whiplash | S13.425A | Sprain of ligaments of cervical spine, initial encounter |
| 2 | 847 | Sprain/whiplash | S13.426A | Sprain of ligaments of cervical spine, initial encounter |
| 2 | 847 | Sprain/whiplash | S13.427A | Sprain of ligaments of cervical spine, initial encounter |
| 2 | 847 | Sprain/whiplash | S13.428A | Sprain of ligaments of cervical spine, initial encounter |
| 2 | 847 | Sprain/whiplash | S13.429A | Sprain of ligaments of cervical spine, initial encounter |
| 2 | 847 | Sprain/whiplash | S13.430A | Sprain of ligaments of cervical spine, initial encounter |
| 2 | 847 | Sprain/whiplash | S13.431A | Sprain of ligaments of cervical spine, initial encounter |
| 2 | 847 | Sprain/whiplash | S13.432A | Sprain of ligaments of cervical spine, initial encounter |
| 2 | 847 | Sprain/whiplash | S13.433A | Sprain of ligaments of cervical spine, initial encounter |
| 2 | 847 | Sprain/whiplash | S13.434A | Sprain of ligaments of cervical spine, initial encounter |
| 2 | 847 | Sprain/whiplash | S13.435A | Sprain of ligaments of cervical spine, initial encounter |
| 2 | 847 | Sprain/whiplash | S13.436A | Sprain of ligaments of cervical spine, initial encounter |
| 2 | 847 | Sprain/whiplash | S13.437A | Sprain of ligaments of cervical spine, initial encounter |
| 2 | 847 | Sprain/whiplash | S13.438A | Sprain of ligaments of cervical spine, initial encounter |
| 2 | 847 | Sprain/whiplash | S13.439A | Sprain of ligaments of cervical spine, initial encounter |
| 2 | 847 | Sprain/whiplash | S13.440A | Sprain of ligaments of cervical spine, initial encounter |
| 2 | 847 | Sprain/whiplash | S13.441A | Sprain of ligaments of cervical spine, initial encounter |
| 2 | 847 | Sprain/whiplash | S13.442A | Sprain of ligaments of cervical spine, initial encounter |
| 2 | 847 | Sprain/whiplash | S13.443A | Sprain of ligaments of cervical spine, initial encounter |
| 2 | 847 | Sprain/whiplash | S13.444A | Sprain of ligaments of cervical spine, initial encounter |
| 2 | 847 | Sprain/whiplash | S13.445A | Sprain of ligaments of cervical spine, initial encounter |
| 2 | 847 | Sprain/whiplash | S13.446A | Sprain of ligaments of cervical spine, initial encounter |
| 2 | 847 | Sprain/whiplash | S13.447A | Sprain of ligaments of cervical spine, initial encounter |
| 2 | 847 | Sprain/whiplash | S13.448A | Sprain of ligaments of cervical spine, initial encounter |
| 2 | 847 | Sprain/whiplash | S13.449A | Sprain of ligaments of cervical spine, initial encounter |
| 2 | 847 | Sprain/whiplash | S13.450A | Sprain of ligaments of cervical spine, initial encounter |
| 2 | 847 | Sprain/whiplash | S13.451A | Sprain of ligaments of cervical spine, initial encounter |
| 2 | 847 | Sprain/whiplash | S13.452A | Sprain of ligaments of cervical spine, initial encounter |
| 2 | 847 | Sprain/whiplash | S13.453A | Sprain of ligaments of cervical spine, initial encounter |
| 2 | 847 | Sprain/whiplash | S13.454A | Sprain of ligaments of cervical spine, initial encounter |
| 2 | 847 | Sprain/whiplash | S13.455A | Sprain of ligaments of cervical spine, initial encounter |
| 2 | 847 | Sprain/whiplash | S13.456A | Sprain of ligaments of cervical spine, initial encounter |
| 2 | 847 | Sprain/whiplash | S13.457A | Sprain of ligaments of cervical spine, initial encounter |
| 2 | 847 | Sprain/whiplash | S13.458A | Sprain of ligaments of cervical spine, initial encounter |
| 2 | 847 | Sprain/whiplash | S13.459A | Sprain of ligaments of cervical spine, initial encounter |
| 2 | 847 | Sprain/whiplash | S13.460A | Sprain of ligaments of cervical spine, initial encounter |
| 2 | 847 | Sprain/whiplash | S13.461A | Sprain of ligaments of cervical spine, initial encounter |
| 2 | 847 | Sprain/whiplash | S13.462A | Sprain of ligaments of cervical spine, initial encounter |
| 2 | 847 | Sprain/whiplash | S13.463A | Sprain of ligaments of cervical spine, initial encounter |
| 2 | 847 | Sprain/whiplash | S13.464A | Sprain of ligaments of cervical spine, initial encounter |
| 2 | 847 | Sprain/whiplash | S13.465A | Sprain of ligaments of cervical spine, initial encounter |
| 2 | 847 | Sprain/whiplash | S13.466A | Sprain of ligaments of cervical spine, initial encounter |
| 2 | 847 | Sprain/whiplash | S13.467A | Sprain of ligaments of cervical spine, initial encounter |
| 2 | 847 | Sprain/whiplash | S13.468A | Sprain of ligaments of cervical spine, initial encounter |
| 2 | 847 | Sprain/whiplash | S13.469A | Sprain of ligaments of cervical spine, initial encounter |
| 2 | 847 | Sprain/whiplash | S13.470A | Sprain of ligaments of cervical spine, initial encounter |
| 2 | 847 | Sprain/whiplash | S13.471A | Sprain of ligaments of cervical spine, initial encounter |
| 2 | 847 | Sprain/whiplash | S13.472A | Sprain of ligaments of cervical spine, initial encounter |
| 2 | 847 | Sprain/whiplash | S13.473A | Sprain of ligaments of cervical spine, initial encounter |
| 2 | 847 | Sprain/whiplash | S13.474A | Sprain of ligaments of cervical spine, initial encounter |
| 2 | 847 | Sprain/whiplash | S13.475A | Sprain of ligaments of cervical spine, initial encounter |
| 2 | 847 | Sprain/whiplash | S13.476A | Sprain of ligaments of cervical spine, initial encounter |
| 2 | 847 | Sprain/whiplash | S13.477A | Sprain of ligaments of cervical spine, initial encounter |
| 2 | 847 | Sprain/whiplash | S13.478A | Sprain of ligaments of cervical spine, initial encounter |
| 2 | 847 | Sprain/whiplash | S13.479A | Sprain of ligaments of cervical spine, initial encounter |
| 2 | 847 | Sprain/whiplash | S13.480A | Sprain of ligaments of cervical spine, initial encounter |
| 2 | 847 | Sprain/whiplash | S13.481A | Sprain of ligaments of cervical spine, initial encounter |
| 2 | 847 | Sprain/whiplash | S13.482A | Sprain of ligaments of cervical spine, initial encounter |
| 2 | 847 | Sprain/whiplash | S13.483A | Sprain of ligaments of cervical spine, initial encounter |
| 2 | 847 | Sprain/whiplash | S13.484A | Sprain of ligaments of cervical spine, initial encounter |
| 2 | 847 | Sprain/whiplash | S13.485A | Sprain of ligaments of cervical spine, initial encounter |
| 2 | 847 | Sprain/whiplash | S13.486A | Sprain of ligaments of cervical spine, initial encounter |
| 2 | 847 | Sprain/whiplash | S13.487A | Sprain of ligaments of cervical spine, initial encounter |
| 2 | 847 | Sprain/whiplash | S13.488A | Sprain of ligaments of cervical spine, initial encounter |
| 2 | 847 | Sprain/whiplash | S13.489A | Sprain of ligaments of cervical spine, initial encounter |
| 2 | 847 | Sprain/whiplash | S13.490A | Sprain of ligaments of cervical spine, initial encounter |
| 2 | 847 | Sprain/whiplash | S13.491A | Sprain of ligaments of cervical spine, initial encounter |
| 2 | 847 | Sprain/whiplash | S13.492A | Sprain of ligaments of cervical spine, initial encounter |
| 2 | 847 | Sprain/whiplash | S13.493A | Sprain of ligaments of cervical spine, initial encounter |
| 2 | 847 | Sprain/whiplash | S13.494A | Sprain of ligaments of cervical spine, initial encounter |
| 2 | 847 | Sprain/whiplash | S13.495A | Sprain of ligaments of cervical spine, initial encounter |
| 2 | 847 | Sprain/whiplash | S13.496A | Sprain of ligaments of cervical spine, initial encounter |
| 2 | 847 | Sprain/whiplash | S13.497A | Sprain of ligaments of cervical spine, initial encounter |
| 2 | 847 | Sprain/whiplash | S13.498A | Sprain of ligaments of cervical spine, initial encounter |
| 2 | 847 | Sprain/whiplash | S13.499A | Sprain of ligaments of cervical spine, initial encounter |
| 2 | 847 | Sprain/whiplash | S13.800A | Sprain of joints and ligaments of other parts of neck, initial encounter |
| 2 | 847 | Sprain/whiplash | S13.801A | Sprain of joints and ligaments of other parts of neck, initial encounter |
| 2 | 847 | Sprain/whiplash | S13.802A | Sprain of joints and ligaments of other parts of neck, initial encounter |
| 2 | 847 | Sprain/whiplash | S13.803A | Sprain of joints and ligaments of other parts of neck, initial encounter |
| 2 | 847 | Sprain/whiplash | S13.804A | Sprain of joints and ligaments of other parts of neck, initial encounter |
| 2 | 847 | Sprain/whiplash | S13.805A | Sprain of joints and ligaments of other parts of neck, initial encounter |
| 2 | 847 | Sprain/whiplash | S13.806A | Sprain of joints and ligaments of other parts of neck, initial encounter |
| 2 | 847 | Sprain/whiplash | S13.807A | Sprain of joints and ligaments of other parts of neck, initial encounter |
| 2 | 847 | Sprain/whiplash | S13.808A | Sprain of joints and ligaments of other parts of neck, initial encounter |
| 2 | 847 | Sprain/whiplash | S13.809A | Sprain of joints and ligaments of other parts of neck, initial encounter |
| 2 | 847 | Sprain/whiplash | S13.810A | Sprain of joints and ligaments of other parts of neck, initial encounter |
| 2 | 847 | Sprain/whiplash | S13.811A | Sprain of joints and ligaments of other parts of neck, initial encounter |
| 2 | 847 | Sprain/whiplash | S13.812A | Sprain of joints and ligaments of other parts of neck, initial encounter |
| 2 | 847 | Sprain/whiplash | S13.813A | Sprain of joints and ligaments of other parts of neck, initial encounter |
| 2 | 847 | Sprain/whiplash | S13.814A | Sprain of joints and ligaments of other parts of neck, initial encounter |
| 2 | 847 | Sprain/whiplash | S13.815A | Sprain of joints and ligaments of other parts of neck, initial encounter |
| 2 | 847 | Sprain/whiplash | S13.816A | Sprain of joints and ligaments of other parts of neck, initial encounter |
| 2 | 847 | Sprain/whiplash | S13.817A | Sprain of joints and ligaments of other parts of neck, initial encounter |
| 2 | 847 | Sprain/whiplash | S13.818A | Sprain of joints and ligaments of other parts of neck, initial encounter |
| 2 | 847 | Sprain/whiplash | S13.819A | Sprain of joints and ligaments of other parts of neck, initial encounter |
| 2 | 847 | Sprain/whiplash | S13.820A | Sprain of joints and ligaments of other parts of neck, initial encounter |
| 2 | 847 | Sprain/whiplash | S13.821A | Sprain of joints and ligaments of other parts of neck, initial encounter |
| 2 | 847 | Sprain/whiplash | S13.822A | Sprain of joints and ligaments of other parts of neck, initial encounter |
| 2 | 847 | Sprain/whiplash | S13.823A | Sprain of joints and ligaments of other parts of neck, initial encounter |
| 2 | 847 | Sprain/whiplash | S13.824A | Sprain of joints and ligaments of other parts of neck, initial encounter |
| 2 | 847 | Sprain/whiplash | S13.825A | Sprain of joints and ligaments of other parts of neck, initial encounter |
| 2 | 847 | Sprain/whiplash | S13.826A | Sprain of joints and ligaments of other parts of neck, initial encounter |
| 2 | 847 | Sprain/whiplash | S13.827A | Sprain of joints and ligaments of other parts of neck, initial encounter |
| 2 | 847 | Sprain/whiplash | S13.828A | Sprain of joints and ligaments of other parts of neck, initial encounter |
| 2 | 847 | Sprain/whiplash | S13.829A | Sprain of joints and ligaments of other parts of neck, initial encounter |
| 2 | 847 | Sprain/whiplash | S13.830A | Sprain of joints and ligaments of other parts of neck, initial encounter |
| 2 | 847 | Sprain/whiplash | S13.831A | Sprain of joints and ligaments of other parts of neck, initial encounter |
| 2 | 847 | Sprain/whiplash | S13.832A | Sprain of joints and ligaments of other parts of neck, initial encounter |
| 2 | 847 | Sprain/whiplash | S13.833A | Sprain of joints and ligaments of other parts of neck, initial encounter |
| 2 | 847 | Sprain/whiplash | S13.834A | Sprain of joints and ligaments of other parts of neck, initial encounter |
| 2 | 847 | Sprain/whiplash | S13.835A | Sprain of joints and ligaments of other parts of neck, initial encounter |
| 2 | 847 | Sprain/whiplash | S13.836A | Sprain of joints and ligaments of other parts of neck, initial encounter |
| 2 | 847 | Sprain/whiplash | S13.837A | Sprain of joints and ligaments of other parts of neck, initial encounter |
| 2 | 847 | Sprain/whiplash | S13.838A | Sprain of joints and ligaments of other parts of neck, initial encounter |
| 2 | 847 | Sprain/whiplash | S13.839A | Sprain of joints and ligaments of other parts of neck, initial encounter |
| 2 | 847 | Sprain/whiplash | S13.840A | Sprain of joints and ligaments of other parts of neck, initial encounter |
| 2 | 847 | Sprain/whiplash | S13.841A | Sprain of joints and ligaments of other parts of neck, initial encounter |
| 2 | 847 | Sprain/whiplash | S13.842A | Sprain of joints and ligaments of other parts of neck, initial encounter |
| 2 | 847 | Sprain/whiplash | S13.843A | Sprain of joints and ligaments of other parts of neck, initial encounter |
| 2 | 847 | Sprain/whiplash | S13.844A | Sprain of joints and ligaments of other parts of neck, initial encounter |
| 2 | 847 | Sprain/whiplash | S13.845A | Sprain of joints and ligaments of other parts of neck, initial encounter |
| 2 | 847 | Sprain/whiplash | S13.846A | Sprain of joints and ligaments of other parts of neck, initial encounter |
| 2 | 847 | Sprain/whiplash | S13.847A | Sprain of joints and ligaments of other parts of neck, initial encounter |
| 2 | 847 | Sprain/whiplash | S13.848A | Sprain of joints and ligaments of other parts of neck, initial encounter |
| 2 | 847 | Sprain/whiplash | S13.849A | Sprain of joints and ligaments of other parts of neck, initial encounter |
| 2 | 847 | Sprain/whiplash | S13.850A | Sprain of joints and ligaments of other parts of neck, initial encounter |
| 2 | 847 | Sprain/whiplash | S13.851A | Sprain of joints and ligaments of other parts of neck, initial encounter |
| 2 | 847 | Sprain/whiplash | S13.852A | Sprain of joints and ligaments of other parts of neck, initial encounter |
| 2 | 847 | Sprain/whiplash | S13.853A | Sprain of joints and ligaments of other parts of neck, initial encounter |
| 2 | 847 | Sprain/whiplash | S13.854A | Sprain of joints and ligaments of other parts of neck, initial encounter |
| 2 | 847 | Sprain/whiplash | S13.855A | Sprain of joints and ligaments of other parts of neck, initial encounter |
| 2 | 847 | Sprain/whiplash | S13.856A | Sprain of joints and ligaments of other parts of neck, initial encounter |
| 2 | 847 | Sprain/whiplash | S13.857A | Sprain of joints and ligaments of other parts of neck, initial encounter |
| 2 | 847 | Sprain/whiplash | S13.858A | Sprain of joints and ligaments of other parts of neck, initial encounter |
| 2 | 847 | Sprain/whiplash | S13.859A | Sprain of joints and ligaments of other parts of neck, initial encounter |
| 2 | 847 | Sprain/whiplash | S13.860A | Sprain of joints and ligaments of other parts of neck, initial encounter |
| 2 | 847 | Sprain/whiplash | S13.861A | Sprain of joints and ligaments of other parts of neck, initial encounter |
| 2 | 847 | Sprain/whiplash | S13.862A | Sprain of joints and ligaments of other parts of neck, initial encounter |
| 2 | 847 | Sprain/whiplash | S13.863A | Sprain of joints and ligaments of other parts of neck, initial encounter |
| 2 | 847 | Sprain/whiplash | S13.864A | Sprain of joints and ligaments of other parts of neck, initial encounter |
| 2 | 847 | Sprain/whiplash | S13.865A | Sprain of joints and ligaments of other parts of neck, initial encounter |
| 2 | 847 | Sprain/whiplash | S13.866A | Sprain of joints and ligaments of other parts of neck, initial encounter |
| 2 | 847 | Sprain/whiplash | S13.867A | Sprain of joints and ligaments of other parts of neck, initial encounter |
| 2 | 847 | Sprain/whiplash | S13.868A | Sprain of joints and ligaments of other parts of neck, initial encounter |
| 2 | 847 | Sprain/whiplash | S13.869A | Sprain of joints and ligaments of other parts of neck, initial encounter |
| 2 | 847 | Sprain/whiplash | S13.870A | Sprain of joints and ligaments of other parts of neck, initial encounter |
| 2 | 847 | Sprain/whiplash | S13.871A | Sprain of joints and ligaments of other parts of neck, initial encounter |
| 2 | 847 | Sprain/whiplash | S13.872A | Sprain of joints and ligaments of other parts of neck, initial encounter |
| 2 | 847 | Sprain/whiplash | S13.873A | Sprain of joints and ligaments of other parts of neck, initial encounter |
| 2 | 847 | Sprain/whiplash | S13.874A | Sprain of joints and ligaments of other parts of neck, initial encounter |
| 2 | 847 | Sprain/whiplash | S13.875A | Sprain of joints and ligaments of other parts of neck, initial encounter |
| 2 | 847 | Sprain/whiplash | S13.876A | Sprain of joints and ligaments of other parts of neck, initial encounter |
| 2 | 847 | Sprain/whiplash | S13.877A | Sprain of joints and ligaments of other parts of neck, initial encounter |
| 2 | 847 | Sprain/whiplash | S13.878A | Sprain of joints and ligaments of other parts of neck, initial encounter |
| 2 | 847 | Sprain/whiplash | S13.879A | Sprain of joints and ligaments of other parts of neck, initial encounter |
| 2 | 847 | Sprain/whiplash | S13.880A | Sprain of joints and ligaments of other parts of neck, initial encounter |
| 2 | 847 | Sprain/whiplash | S13.881A | Sprain of joints and ligaments of other parts of neck, initial encounter |
| 2 | 847 | Sprain/whiplash | S13.882A | Sprain of joints and ligaments of other parts of neck, initial encounter |
| 2 | 847 | Sprain/whiplash | S13.883A | Sprain of joints and ligaments of other parts of neck, initial encounter |
| 2 | 847 | Sprain/whiplash | S13.884A | Sprain of joints and ligaments of other parts of neck, initial encounter |
| 2 | 847 | Sprain/whiplash | S13.885A | Sprain of joints and ligaments of other parts of neck, initial encounter |
| 2 | 847 | Sprain/whiplash | S13.886A | Sprain of joints and ligaments of other parts of neck, initial encounter |
| 2 | 847 | Sprain/whiplash | S13.887A | Sprain of joints and ligaments of other parts of neck, initial encounter |
| 2 | 847 | Sprain/whiplash | S13.888A | Sprain of joints and ligaments of other parts of neck, initial encounter |
| 2 | 847 | Sprain/whiplash | S13.889A | Sprain of joints and ligaments of other parts of neck, initial encounter |
| 2 | 847 | Sprain/whiplash | S13.890A | Sprain of joints and ligaments of other parts of neck, initial encounter |
| 2 | 847 | Sprain/whiplash | S13.891A | Sprain of joints and ligaments of other parts of neck, initial encounter |
| 2 | 847 | Sprain/whiplash | S13.892A | Sprain of joints and ligaments of other parts of neck, initial encounter |
| 2 | 847 | Sprain/whiplash | S13.893A | Sprain of joints and ligaments of other parts of neck, initial encounter |
| 2 | 847 | Sprain/whiplash | S13.894A | Sprain of joints and ligaments of other parts of neck, initial encounter |
| 2 | 847 | Sprain/whiplash | S13.895A | Sprain of joints and ligaments of other parts of neck, initial encounter |
| 2 | 847 | Sprain/whiplash | S13.896A | Sprain of joints and ligaments of other parts of neck, initial encounter |
| 2 | 847 | Sprain/whiplash | S13.897A | Sprain of joints and ligaments of other parts of neck, initial encounter |
| 2 | 847 | Sprain/whiplash | S13.898A | Sprain of joints and ligaments of other parts of neck, initial encounter |
| 2 | 847 | Sprain/whiplash | S13.899A | Sprain of joints and ligaments of other parts of neck, initial encounter |
| 2 | 847.1 | Sprain | S23.300A | Sprain of ligaments of thoracic spine, initial encounter |
| 2 | 847.1 | Sprain | S23.301A | Sprain of ligaments of thoracic spine, initial encounter |
| 2 | 847.1 | Sprain | S23.302A | Sprain of ligaments of thoracic spine, initial encounter |
| 2 | 847.1 | Sprain | S23.303A | Sprain of ligaments of thoracic spine, initial encounter |
| 2 | 847.1 | Sprain | S23.304A | Sprain of ligaments of thoracic spine, initial encounter |
| 2 | 847.1 | Sprain | S23.305A | Sprain of ligaments of thoracic spine, initial encounter |
| 2 | 847.1 | Sprain | S23.306A | Sprain of ligaments of thoracic spine, initial encounter |
| 2 | 847.1 | Sprain | S23.307A | Sprain of ligaments of thoracic spine, initial encounter |
| 2 | 847.1 | Sprain | S23.308A | Sprain of ligaments of thoracic spine, initial encounter |
| 2 | 847.1 | Sprain | S23.309A | Sprain of ligaments of thoracic spine, initial encounter |
| 2 | 847.1 | Sprain | S23.310A | Sprain of ligaments of thoracic spine, initial encounter |
| 2 | 847.1 | Sprain | S23.311A | Sprain of ligaments of thoracic spine, initial encounter |
| 2 | 847.1 | Sprain | S23.312A | Sprain of ligaments of thoracic spine, initial encounter |
| 2 | 847.1 | Sprain | S23.313A | Sprain of ligaments of thoracic spine, initial encounter |
| 2 | 847.1 | Sprain | S23.314A | Sprain of ligaments of thoracic spine, initial encounter |
| 2 | 847.1 | Sprain | S23.315A | Sprain of ligaments of thoracic spine, initial encounter |
| 2 | 847.1 | Sprain | S23.316A | Sprain of ligaments of thoracic spine, initial encounter |
| 2 | 847.1 | Sprain | S23.317A | Sprain of ligaments of thoracic spine, initial encounter |
| 2 | 847.1 | Sprain | S23.318A | Sprain of ligaments of thoracic spine, initial encounter |
| 2 | 847.1 | Sprain | S23.319A | Sprain of ligaments of thoracic spine, initial encounter |
| 2 | 847.1 | Sprain | S23.320A | Sprain of ligaments of thoracic spine, initial encounter |
| 2 | 847.1 | Sprain | S23.321A | Sprain of ligaments of thoracic spine, initial encounter |
| 2 | 847.1 | Sprain | S23.322A | Sprain of ligaments of thoracic spine, initial encounter |
| 2 | 847.1 | Sprain | S23.323A | Sprain of ligaments of thoracic spine, initial encounter |
| 2 | 847.1 | Sprain | S23.324A | Sprain of ligaments of thoracic spine, initial encounter |
| 2 | 847.1 | Sprain | S23.325A | Sprain of ligaments of thoracic spine, initial encounter |
| 2 | 847.1 | Sprain | S23.326A | Sprain of ligaments of thoracic spine, initial encounter |
| 2 | 847.1 | Sprain | S23.327A | Sprain of ligaments of thoracic spine, initial encounter |
| 2 | 847.1 | Sprain | S23.328A | Sprain of ligaments of thoracic spine, initial encounter |
| 2 | 847.1 | Sprain | S23.329A | Sprain of ligaments of thoracic spine, initial encounter |
| 2 | 847.1 | Sprain | S23.330A | Sprain of ligaments of thoracic spine, initial encounter |
| 2 | 847.1 | Sprain | S23.331A | Sprain of ligaments of thoracic spine, initial encounter |
| 2 | 847.1 | Sprain | S23.332A | Sprain of ligaments of thoracic spine, initial encounter |
| 2 | 847.1 | Sprain | S23.333A | Sprain of ligaments of thoracic spine, initial encounter |
| 2 | 847.1 | Sprain | S23.334A | Sprain of ligaments of thoracic spine, initial encounter |
| 2 | 847.1 | Sprain | S23.335A | Sprain of ligaments of thoracic spine, initial encounter |
| 2 | 847.1 | Sprain | S23.336A | Sprain of ligaments of thoracic spine, initial encounter |
| 2 | 847.1 | Sprain | S23.337A | Sprain of ligaments of thoracic spine, initial encounter |
| 2 | 847.1 | Sprain | S23.338A | Sprain of ligaments of thoracic spine, initial encounter |
| 2 | 847.1 | Sprain | S23.339A | Sprain of ligaments of thoracic spine, initial encounter |
| 2 | 847.1 | Sprain | S23.340A | Sprain of ligaments of thoracic spine, initial encounter |
| 2 | 847.1 | Sprain | S23.341A | Sprain of ligaments of thoracic spine, initial encounter |
| 2 | 847.1 | Sprain | S23.342A | Sprain of ligaments of thoracic spine, initial encounter |
| 2 | 847.1 | Sprain | S23.343A | Sprain of ligaments of thoracic spine, initial encounter |
| 2 | 847.1 | Sprain | S23.344A | Sprain of ligaments of thoracic spine, initial encounter |
| 2 | 847.1 | Sprain | S23.345A | Sprain of ligaments of thoracic spine, initial encounter |
| 2 | 847.1 | Sprain | S23.346A | Sprain of ligaments of thoracic spine, initial encounter |
| 2 | 847.1 | Sprain | S23.347A | Sprain of ligaments of thoracic spine, initial encounter |
| 2 | 847.1 | Sprain | S23.348A | Sprain of ligaments of thoracic spine, initial encounter |
| 2 | 847.1 | Sprain | S23.349A | Sprain of ligaments of thoracic spine, initial encounter |
| 2 | 847.1 | Sprain | S23.350A | Sprain of ligaments of thoracic spine, initial encounter |
| 2 | 847.1 | Sprain | S23.351A | Sprain of ligaments of thoracic spine, initial encounter |
| 2 | 847.1 | Sprain | S23.352A | Sprain of ligaments of thoracic spine, initial encounter |
| 2 | 847.1 | Sprain | S23.353A | Sprain of ligaments of thoracic spine, initial encounter |
| 2 | 847.1 | Sprain | S23.354A | Sprain of ligaments of thoracic spine, initial encounter |
| 2 | 847.1 | Sprain | S23.355A | Sprain of ligaments of thoracic spine, initial encounter |
| 2 | 847.1 | Sprain | S23.356A | Sprain of ligaments of thoracic spine, initial encounter |
| 2 | 847.1 | Sprain | S23.357A | Sprain of ligaments of thoracic spine, initial encounter |
| 2 | 847.1 | Sprain | S23.358A | Sprain of ligaments of thoracic spine, initial encounter |
| 2 | 847.1 | Sprain | S23.359A | Sprain of ligaments of thoracic spine, initial encounter |
| 2 | 847.1 | Sprain | S23.360A | Sprain of ligaments of thoracic spine, initial encounter |
| 2 | 847.1 | Sprain | S23.361A | Sprain of ligaments of thoracic spine, initial encounter |
| 2 | 847.1 | Sprain | S23.362A | Sprain of ligaments of thoracic spine, initial encounter |
| 2 | 847.1 | Sprain | S23.363A | Sprain of ligaments of thoracic spine, initial encounter |
| 2 | 847.1 | Sprain | S23.364A | Sprain of ligaments of thoracic spine, initial encounter |
| 2 | 847.1 | Sprain | S23.365A | Sprain of ligaments of thoracic spine, initial encounter |
| 2 | 847.1 | Sprain | S23.366A | Sprain of ligaments of thoracic spine, initial encounter |
| 2 | 847.1 | Sprain | S23.367A | Sprain of ligaments of thoracic spine, initial encounter |
| 2 | 847.1 | Sprain | S23.368A | Sprain of ligaments of thoracic spine, initial encounter |
| 2 | 847.1 | Sprain | S23.369A | Sprain of ligaments of thoracic spine, initial encounter |
| 2 | 847.1 | Sprain | S23.370A | Sprain of ligaments of thoracic spine, initial encounter |
| 2 | 847.1 | Sprain | S23.371A | Sprain of ligaments of thoracic spine, initial encounter |
| 2 | 847.1 | Sprain | S23.372A | Sprain of ligaments of thoracic spine, initial encounter |
| 2 | 847.1 | Sprain | S23.373A | Sprain of ligaments of thoracic spine, initial encounter |
| 2 | 847.1 | Sprain | S23.374A | Sprain of ligaments of thoracic spine, initial encounter |
| 2 | 847.1 | Sprain | S23.375A | Sprain of ligaments of thoracic spine, initial encounter |
| 2 | 847.1 | Sprain | S23.376A | Sprain of ligaments of thoracic spine, initial encounter |
| 2 | 847.1 | Sprain | S23.377A | Sprain of ligaments of thoracic spine, initial encounter |
| 2 | 847.1 | Sprain | S23.378A | Sprain of ligaments of thoracic spine, initial encounter |
| 2 | 847.1 | Sprain | S23.379A | Sprain of ligaments of thoracic spine, initial encounter |
| 2 | 847.1 | Sprain | S23.380A | Sprain of ligaments of thoracic spine, initial encounter |
| 2 | 847.1 | Sprain | S23.381A | Sprain of ligaments of thoracic spine, initial encounter |
| 2 | 847.1 | Sprain | S23.382A | Sprain of ligaments of thoracic spine, initial encounter |
| 2 | 847.1 | Sprain | S23.383A | Sprain of ligaments of thoracic spine, initial encounter |
| 2 | 847.1 | Sprain | S23.384A | Sprain of ligaments of thoracic spine, initial encounter |
| 2 | 847.1 | Sprain | S23.385A | Sprain of ligaments of thoracic spine, initial encounter |
| 2 | 847.1 | Sprain | S23.386A | Sprain of ligaments of thoracic spine, initial encounter |
| 2 | 847.1 | Sprain | S23.387A | Sprain of ligaments of thoracic spine, initial encounter |
| 2 | 847.1 | Sprain | S23.388A | Sprain of ligaments of thoracic spine, initial encounter |
| 2 | 847.1 | Sprain | S23.389A | Sprain of ligaments of thoracic spine, initial encounter |
| 2 | 847.1 | Sprain | S23.390A | Sprain of ligaments of thoracic spine, initial encounter |
| 2 | 847.1 | Sprain | S23.391A | Sprain of ligaments of thoracic spine, initial encounter |
| 2 | 847.1 | Sprain | S23.392A | Sprain of ligaments of thoracic spine, initial encounter |
| 2 | 847.1 | Sprain | S23.393A | Sprain of ligaments of thoracic spine, initial encounter |
| 2 | 847.1 | Sprain | S23.394A | Sprain of ligaments of thoracic spine, initial encounter |
| 2 | 847.1 | Sprain | S23.395A | Sprain of ligaments of thoracic spine, initial encounter |
| 2 | 847.1 | Sprain | S23.396A | Sprain of ligaments of thoracic spine, initial encounter |
| 2 | 847.1 | Sprain | S23.397A | Sprain of ligaments of thoracic spine, initial encounter |
| 2 | 847.1 | Sprain | S23.398A | Sprain of ligaments of thoracic spine, initial encounter |
| 2 | 847.1 | Sprain | S23.399A | Sprain of ligaments of thoracic spine, initial encounter |
| 2 | 847.1 | Sprain | S23.800A | Sprain of other unspecified parts of thorax, initial encounter |
| 2 | 847.1 | Sprain | S23.801A | Sprain of other unspecified parts of thorax, initial encounter |
| 2 | 847.1 | Sprain | S23.802A | Sprain of other unspecified parts of thorax, initial encounter |
| 2 | 847.1 | Sprain | S23.803A | Sprain of other unspecified parts of thorax, initial encounter |
| 2 | 847.1 | Sprain | S23.804A | Sprain of other unspecified parts of thorax, initial encounter |
| 2 | 847.1 | Sprain | S23.805A | Sprain of other unspecified parts of thorax, initial encounter |
| 2 | 847.1 | Sprain | S23.806A | Sprain of other unspecified parts of thorax, initial encounter |
| 2 | 847.1 | Sprain | S23.807A | Sprain of other unspecified parts of thorax, initial encounter |
| 2 | 847.1 | Sprain | S23.808A | Sprain of other unspecified parts of thorax, initial encounter |
| 2 | 847.1 | Sprain | S23.809A | Sprain of other unspecified parts of thorax, initial encounter |
| 2 | 847.1 | Sprain | S23.810A | Sprain of other unspecified parts of thorax, initial encounter |
| 2 | 847.1 | Sprain | S23.811A | Sprain of other unspecified parts of thorax, initial encounter |
| 2 | 847.1 | Sprain | S23.812A | Sprain of other unspecified parts of thorax, initial encounter |
| 2 | 847.1 | Sprain | S23.813A | Sprain of other unspecified parts of thorax, initial encounter |
| 2 | 847.1 | Sprain | S23.814A | Sprain of other unspecified parts of thorax, initial encounter |
| 2 | 847.1 | Sprain | S23.815A | Sprain of other unspecified parts of thorax, initial encounter |
| 2 | 847.1 | Sprain | S23.816A | Sprain of other unspecified parts of thorax, initial encounter |
| 2 | 847.1 | Sprain | S23.817A | Sprain of other unspecified parts of thorax, initial encounter |
| 2 | 847.1 | Sprain | S23.818A | Sprain of other unspecified parts of thorax, initial encounter |
| 2 | 847.1 | Sprain | S23.819A | Sprain of other unspecified parts of thorax, initial encounter |
| 2 | 847.1 | Sprain | S23.820A | Sprain of other unspecified parts of thorax, initial encounter |
| 2 | 847.1 | Sprain | S23.821A | Sprain of other unspecified parts of thorax, initial encounter |
| 2 | 847.1 | Sprain | S23.822A | Sprain of other unspecified parts of thorax, initial encounter |
| 2 | 847.1 | Sprain | S23.823A | Sprain of other unspecified parts of thorax, initial encounter |
| 2 | 847.1 | Sprain | S23.824A | Sprain of other unspecified parts of thorax, initial encounter |
| 2 | 847.1 | Sprain | S23.825A | Sprain of other unspecified parts of thorax, initial encounter |
| 2 | 847.1 | Sprain | S23.826A | Sprain of other unspecified parts of thorax, initial encounter |
| 2 | 847.1 | Sprain | S23.827A | Sprain of other unspecified parts of thorax, initial encounter |
| 2 | 847.1 | Sprain | S23.828A | Sprain of other unspecified parts of thorax, initial encounter |
| 2 | 847.1 | Sprain | S23.829A | Sprain of other unspecified parts of thorax, initial encounter |
| 2 | 847.1 | Sprain | S23.830A | Sprain of other unspecified parts of thorax, initial encounter |
| 2 | 847.1 | Sprain | S23.831A | Sprain of other unspecified parts of thorax, initial encounter |
| 2 | 847.1 | Sprain | S23.832A | Sprain of other unspecified parts of thorax, initial encounter |
| 2 | 847.1 | Sprain | S23.833A | Sprain of other unspecified parts of thorax, initial encounter |
| 2 | 847.1 | Sprain | S23.834A | Sprain of other unspecified parts of thorax, initial encounter |
| 2 | 847.1 | Sprain | S23.835A | Sprain of other unspecified parts of thorax, initial encounter |
| 2 | 847.1 | Sprain | S23.836A | Sprain of other unspecified parts of thorax, initial encounter |
| 2 | 847.1 | Sprain | S23.837A | Sprain of other unspecified parts of thorax, initial encounter |
| 2 | 847.1 | Sprain | S23.838A | Sprain of other unspecified parts of thorax, initial encounter |
| 2 | 847.1 | Sprain | S23.839A | Sprain of other unspecified parts of thorax, initial encounter |
| 2 | 847.1 | Sprain | S23.840A | Sprain of other unspecified parts of thorax, initial encounter |
| 2 | 847.1 | Sprain | S23.841A | Sprain of other unspecified parts of thorax, initial encounter |
| 2 | 847.1 | Sprain | S23.842A | Sprain of other unspecified parts of thorax, initial encounter |
| 2 | 847.1 | Sprain | S23.843A | Sprain of other unspecified parts of thorax, initial encounter |
| 2 | 847.1 | Sprain | S23.844A | Sprain of other unspecified parts of thorax, initial encounter |
| 2 | 847.1 | Sprain | S23.845A | Sprain of other unspecified parts of thorax, initial encounter |
| 2 | 847.1 | Sprain | S23.846A | Sprain of other unspecified parts of thorax, initial encounter |
| 2 | 847.1 | Sprain | S23.847A | Sprain of other unspecified parts of thorax, initial encounter |
| 2 | 847.1 | Sprain | S23.848A | Sprain of other unspecified parts of thorax, initial encounter |
| 2 | 847.1 | Sprain | S23.849A | Sprain of other unspecified parts of thorax, initial encounter |
| 2 | 847.1 | Sprain | S23.850A | Sprain of other unspecified parts of thorax, initial encounter |
| 2 | 847.1 | Sprain | S23.851A | Sprain of other unspecified parts of thorax, initial encounter |
| 2 | 847.1 | Sprain | S23.852A | Sprain of other unspecified parts of thorax, initial encounter |
| 2 | 847.1 | Sprain | S23.853A | Sprain of other unspecified parts of thorax, initial encounter |
| 2 | 847.1 | Sprain | S23.854A | Sprain of other unspecified parts of thorax, initial encounter |
| 2 | 847.1 | Sprain | S23.855A | Sprain of other unspecified parts of thorax, initial encounter |
| 2 | 847.1 | Sprain | S23.856A | Sprain of other unspecified parts of thorax, initial encounter |
| 2 | 847.1 | Sprain | S23.857A | Sprain of other unspecified parts of thorax, initial encounter |
| 2 | 847.1 | Sprain | S23.858A | Sprain of other unspecified parts of thorax, initial encounter |
| 2 | 847.1 | Sprain | S23.859A | Sprain of other unspecified parts of thorax, initial encounter |
| 2 | 847.1 | Sprain | S23.860A | Sprain of other unspecified parts of thorax, initial encounter |
| 2 | 847.1 | Sprain | S23.861A | Sprain of other unspecified parts of thorax, initial encounter |
| 2 | 847.1 | Sprain | S23.862A | Sprain of other unspecified parts of thorax, initial encounter |
| 2 | 847.1 | Sprain | S23.863A | Sprain of other unspecified parts of thorax, initial encounter |
| 2 | 847.1 | Sprain | S23.864A | Sprain of other unspecified parts of thorax, initial encounter |
| 2 | 847.1 | Sprain | S23.865A | Sprain of other unspecified parts of thorax, initial encounter |
| 2 | 847.1 | Sprain | S23.866A | Sprain of other unspecified parts of thorax, initial encounter |
| 2 | 847.1 | Sprain | S23.867A | Sprain of other unspecified parts of thorax, initial encounter |
| 2 | 847.1 | Sprain | S23.868A | Sprain of other unspecified parts of thorax, initial encounter |
| 2 | 847.1 | Sprain | S23.869A | Sprain of other unspecified parts of thorax, initial encounter |
| 2 | 847.1 | Sprain | S23.870A | Sprain of other unspecified parts of thorax, initial encounter |
| 2 | 847.1 | Sprain | S23.871A | Sprain of other unspecified parts of thorax, initial encounter |
| 2 | 847.1 | Sprain | S23.872A | Sprain of other unspecified parts of thorax, initial encounter |
| 2 | 847.1 | Sprain | S23.873A | Sprain of other unspecified parts of thorax, initial encounter |
| 2 | 847.1 | Sprain | S23.874A | Sprain of other unspecified parts of thorax, initial encounter |
| 2 | 847.1 | Sprain | S23.875A | Sprain of other unspecified parts of thorax, initial encounter |
| 2 | 847.1 | Sprain | S23.876A | Sprain of other unspecified parts of thorax, initial encounter |
| 2 | 847.1 | Sprain | S23.877A | Sprain of other unspecified parts of thorax, initial encounter |
| 2 | 847.1 | Sprain | S23.878A | Sprain of other unspecified parts of thorax, initial encounter |
| 2 | 847.1 | Sprain | S23.879A | Sprain of other unspecified parts of thorax, initial encounter |
| 2 | 847.1 | Sprain | S23.880A | Sprain of other unspecified parts of thorax, initial encounter |
| 2 | 847.1 | Sprain | S23.881A | Sprain of other unspecified parts of thorax, initial encounter |
| 2 | 847.1 | Sprain | S23.882A | Sprain of other unspecified parts of thorax, initial encounter |
| 2 | 847.1 | Sprain | S23.883A | Sprain of other unspecified parts of thorax, initial encounter |
| 2 | 847.1 | Sprain | S23.884A | Sprain of other unspecified parts of thorax, initial encounter |
| 2 | 847.1 | Sprain | S23.885A | Sprain of other unspecified parts of thorax, initial encounter |
| 2 | 847.1 | Sprain | S23.886A | Sprain of other unspecified parts of thorax, initial encounter |
| 2 | 847.1 | Sprain | S23.887A | Sprain of other unspecified parts of thorax, initial encounter |
| 2 | 847.1 | Sprain | S23.888A | Sprain of other unspecified parts of thorax, initial encounter |
| 2 | 847.1 | Sprain | S23.889A | Sprain of other unspecified parts of thorax, initial encounter |
| 2 | 847.1 | Sprain | S23.890A | Sprain of other unspecified parts of thorax, initial encounter |
| 2 | 847.1 | Sprain | S23.891A | Sprain of other unspecified parts of thorax, initial encounter |
| 2 | 847.1 | Sprain | S23.892A | Sprain of other unspecified parts of thorax, initial encounter |
| 2 | 847.1 | Sprain | S23.893A | Sprain of other unspecified parts of thorax, initial encounter |
| 2 | 847.1 | Sprain | S23.894A | Sprain of other unspecified parts of thorax, initial encounter |
| 2 | 847.1 | Sprain | S23.895A | Sprain of other unspecified parts of thorax, initial encounter |
| 2 | 847.1 | Sprain | S23.896A | Sprain of other unspecified parts of thorax, initial encounter |
| 2 | 847.1 | Sprain | S23.897A | Sprain of other unspecified parts of thorax, initial encounter |
| 2 | 847.1 | Sprain | S23.898A | Sprain of other unspecified parts of thorax, initial encounter |
| 2 | 847.1 | Sprain | S23.899A | Sprain of other unspecified parts of thorax, initial encounter |
| 2 | 847.2 | Sprain | S33.500A | Sprain of ligaments of lumbar spine, initial encounter |
| 2 | 847.2 | Sprain | S33.501A | Sprain of ligaments of lumbar spine, initial encounter |
| 2 | 847.2 | Sprain | S33.502A | Sprain of ligaments of lumbar spine, initial encounter |
| 2 | 847.2 | Sprain | S33.503A | Sprain of ligaments of lumbar spine, initial encounter |
| 2 | 847.2 | Sprain | S33.504A | Sprain of ligaments of lumbar spine, initial encounter |
| 2 | 847.2 | Sprain | S33.505A | Sprain of ligaments of lumbar spine, initial encounter |
| 2 | 847.2 | Sprain | S33.506A | Sprain of ligaments of lumbar spine, initial encounter |
| 2 | 847.2 | Sprain | S33.507A | Sprain of ligaments of lumbar spine, initial encounter |
| 2 | 847.2 | Sprain | S33.508A | Sprain of ligaments of lumbar spine, initial encounter |
| 2 | 847.2 | Sprain | S33.509A | Sprain of ligaments of lumbar spine, initial encounter |
| 2 | 847.2 | Sprain | S33.510A | Sprain of ligaments of lumbar spine, initial encounter |
| 2 | 847.2 | Sprain | S33.511A | Sprain of ligaments of lumbar spine, initial encounter |
| 2 | 847.2 | Sprain | S33.512A | Sprain of ligaments of lumbar spine, initial encounter |
| 2 | 847.2 | Sprain | S33.513A | Sprain of ligaments of lumbar spine, initial encounter |
| 2 | 847.2 | Sprain | S33.514A | Sprain of ligaments of lumbar spine, initial encounter |
| 2 | 847.2 | Sprain | S33.515A | Sprain of ligaments of lumbar spine, initial encounter |
| 2 | 847.2 | Sprain | S33.516A | Sprain of ligaments of lumbar spine, initial encounter |
| 2 | 847.2 | Sprain | S33.517A | Sprain of ligaments of lumbar spine, initial encounter |
| 2 | 847.2 | Sprain | S33.518A | Sprain of ligaments of lumbar spine, initial encounter |
| 2 | 847.2 | Sprain | S33.519A | Sprain of ligaments of lumbar spine, initial encounter |
| 2 | 847.2 | Sprain | S33.520A | Sprain of ligaments of lumbar spine, initial encounter |
| 2 | 847.2 | Sprain | S33.521A | Sprain of ligaments of lumbar spine, initial encounter |
| 2 | 847.2 | Sprain | S33.522A | Sprain of ligaments of lumbar spine, initial encounter |
| 2 | 847.2 | Sprain | S33.523A | Sprain of ligaments of lumbar spine, initial encounter |
| 2 | 847.2 | Sprain | S33.524A | Sprain of ligaments of lumbar spine, initial encounter |
| 2 | 847.2 | Sprain | S33.525A | Sprain of ligaments of lumbar spine, initial encounter |
| 2 | 847.2 | Sprain | S33.526A | Sprain of ligaments of lumbar spine, initial encounter |
| 2 | 847.2 | Sprain | S33.527A | Sprain of ligaments of lumbar spine, initial encounter |
| 2 | 847.2 | Sprain | S33.528A | Sprain of ligaments of lumbar spine, initial encounter |
| 2 | 847.2 | Sprain | S33.529A | Sprain of ligaments of lumbar spine, initial encounter |
| 2 | 847.2 | Sprain | S33.530A | Sprain of ligaments of lumbar spine, initial encounter |
| 2 | 847.2 | Sprain | S33.531A | Sprain of ligaments of lumbar spine, initial encounter |
| 2 | 847.2 | Sprain | S33.532A | Sprain of ligaments of lumbar spine, initial encounter |
| 2 | 847.2 | Sprain | S33.533A | Sprain of ligaments of lumbar spine, initial encounter |
| 2 | 847.2 | Sprain | S33.534A | Sprain of ligaments of lumbar spine, initial encounter |
| 2 | 847.2 | Sprain | S33.535A | Sprain of ligaments of lumbar spine, initial encounter |
| 2 | 847.2 | Sprain | S33.536A | Sprain of ligaments of lumbar spine, initial encounter |
| 2 | 847.2 | Sprain | S33.537A | Sprain of ligaments of lumbar spine, initial encounter |
| 2 | 847.2 | Sprain | S33.538A | Sprain of ligaments of lumbar spine, initial encounter |
| 2 | 847.2 | Sprain | S33.539A | Sprain of ligaments of lumbar spine, initial encounter |
| 2 | 847.2 | Sprain | S33.540A | Sprain of ligaments of lumbar spine, initial encounter |
| 2 | 847.2 | Sprain | S33.541A | Sprain of ligaments of lumbar spine, initial encounter |
| 2 | 847.2 | Sprain | S33.542A | Sprain of ligaments of lumbar spine, initial encounter |
| 2 | 847.2 | Sprain | S33.543A | Sprain of ligaments of lumbar spine, initial encounter |
| 2 | 847.2 | Sprain | S33.544A | Sprain of ligaments of lumbar spine, initial encounter |
| 2 | 847.2 | Sprain | S33.545A | Sprain of ligaments of lumbar spine, initial encounter |
| 2 | 847.2 | Sprain | S33.546A | Sprain of ligaments of lumbar spine, initial encounter |
| 2 | 847.2 | Sprain | S33.547A | Sprain of ligaments of lumbar spine, initial encounter |
| 2 | 847.2 | Sprain | S33.548A | Sprain of ligaments of lumbar spine, initial encounter |
| 2 | 847.2 | Sprain | S33.549A | Sprain of ligaments of lumbar spine, initial encounter |
| 2 | 847.2 | Sprain | S33.550A | Sprain of ligaments of lumbar spine, initial encounter |
| 2 | 847.2 | Sprain | S33.551A | Sprain of ligaments of lumbar spine, initial encounter |
| 2 | 847.2 | Sprain | S33.552A | Sprain of ligaments of lumbar spine, initial encounter |
| 2 | 847.2 | Sprain | S33.553A | Sprain of ligaments of lumbar spine, initial encounter |
| 2 | 847.2 | Sprain | S33.554A | Sprain of ligaments of lumbar spine, initial encounter |
| 2 | 847.2 | Sprain | S33.555A | Sprain of ligaments of lumbar spine, initial encounter |
| 2 | 847.2 | Sprain | S33.556A | Sprain of ligaments of lumbar spine, initial encounter |
| 2 | 847.2 | Sprain | S33.557A | Sprain of ligaments of lumbar spine, initial encounter |
| 2 | 847.2 | Sprain | S33.558A | Sprain of ligaments of lumbar spine, initial encounter |
| 2 | 847.2 | Sprain | S33.559A | Sprain of ligaments of lumbar spine, initial encounter |
| 2 | 847.2 | Sprain | S33.560A | Sprain of ligaments of lumbar spine, initial encounter |
| 2 | 847.2 | Sprain | S33.561A | Sprain of ligaments of lumbar spine, initial encounter |
| 2 | 847.2 | Sprain | S33.562A | Sprain of ligaments of lumbar spine, initial encounter |
| 2 | 847.2 | Sprain | S33.563A | Sprain of ligaments of lumbar spine, initial encounter |
| 2 | 847.2 | Sprain | S33.564A | Sprain of ligaments of lumbar spine, initial encounter |
| 2 | 847.2 | Sprain | S33.565A | Sprain of ligaments of lumbar spine, initial encounter |
| 2 | 847.2 | Sprain | S33.566A | Sprain of ligaments of lumbar spine, initial encounter |
| 2 | 847.2 | Sprain | S33.567A | Sprain of ligaments of lumbar spine, initial encounter |
| 2 | 847.2 | Sprain | S33.568A | Sprain of ligaments of lumbar spine, initial encounter |
| 2 | 847.2 | Sprain | S33.569A | Sprain of ligaments of lumbar spine, initial encounter |
| 2 | 847.2 | Sprain | S33.570A | Sprain of ligaments of lumbar spine, initial encounter |
| 2 | 847.2 | Sprain | S33.571A | Sprain of ligaments of lumbar spine, initial encounter |
| 2 | 847.2 | Sprain | S33.572A | Sprain of ligaments of lumbar spine, initial encounter |
| 2 | 847.2 | Sprain | S33.573A | Sprain of ligaments of lumbar spine, initial encounter |
| 2 | 847.2 | Sprain | S33.574A | Sprain of ligaments of lumbar spine, initial encounter |
| 2 | 847.2 | Sprain | S33.575A | Sprain of ligaments of lumbar spine, initial encounter |
| 2 | 847.2 | Sprain | S33.576A | Sprain of ligaments of lumbar spine, initial encounter |
| 2 | 847.2 | Sprain | S33.577A | Sprain of ligaments of lumbar spine, initial encounter |
| 2 | 847.2 | Sprain | S33.578A | Sprain of ligaments of lumbar spine, initial encounter |
| 2 | 847.2 | Sprain | S33.579A | Sprain of ligaments of lumbar spine, initial encounter |
| 2 | 847.2 | Sprain | S33.580A | Sprain of ligaments of lumbar spine, initial encounter |
| 2 | 847.2 | Sprain | S33.581A | Sprain of ligaments of lumbar spine, initial encounter |
| 2 | 847.2 | Sprain | S33.582A | Sprain of ligaments of lumbar spine, initial encounter |
| 2 | 847.2 | Sprain | S33.583A | Sprain of ligaments of lumbar spine, initial encounter |
| 2 | 847.2 | Sprain | S33.584A | Sprain of ligaments of lumbar spine, initial encounter |
| 2 | 847.2 | Sprain | S33.585A | Sprain of ligaments of lumbar spine, initial encounter |
| 2 | 847.2 | Sprain | S33.586A | Sprain of ligaments of lumbar spine, initial encounter |
| 2 | 847.2 | Sprain | S33.587A | Sprain of ligaments of lumbar spine, initial encounter |
| 2 | 847.2 | Sprain | S33.588A | Sprain of ligaments of lumbar spine, initial encounter |
| 2 | 847.2 | Sprain | S33.589A | Sprain of ligaments of lumbar spine, initial encounter |
| 2 | 847.2 | Sprain | S33.590A | Sprain of ligaments of lumbar spine, initial encounter |
| 2 | 847.2 | Sprain | S33.591A | Sprain of ligaments of lumbar spine, initial encounter |
| 2 | 847.2 | Sprain | S33.592A | Sprain of ligaments of lumbar spine, initial encounter |
| 2 | 847.2 | Sprain | S33.593A | Sprain of ligaments of lumbar spine, initial encounter |
| 2 | 847.2 | Sprain | S33.594A | Sprain of ligaments of lumbar spine, initial encounter |
| 2 | 847.2 | Sprain | S33.595A | Sprain of ligaments of lumbar spine, initial encounter |
| 2 | 847.2 | Sprain | S33.596A | Sprain of ligaments of lumbar spine, initial encounter |
| 2 | 847.2 | Sprain | S33.597A | Sprain of ligaments of lumbar spine, initial encounter |
| 2 | 847.2 | Sprain | S33.598A | Sprain of ligaments of lumbar spine, initial encounter |
| 2 | 847.2 | Sprain | S33.599A | Sprain of ligaments of lumbar spine, initial encounter |
| 3 | 718.88 | Instability | M24.80 | Other specific joint derangements of unspecified joint |
| 3 | 720 | Inflammatory/Ankylosing spondylosis | M45.9 | Ankylosing spondylitis of unspecified sites in spine |
| 3 | 721.1 | Spondylosis w/myelopathy | M47.12 | Other spondylosis with myelopathy, cervical region |
| 3 | 721.41 | Spondylosis w/myelopathy | M47.14 | Other spondylosis with myelopathy, thoracic region |
| 3 | 721.42 | Spondylosis w/myelopathy | M47.16 | Other spondylosis with myelopathy, lumbar region |
| 3 | 721.8 | DISH | M48.9 | Spondylopathy, unspecified |
| 3 | 722.71 | Disc herniation w/myelopathy | M50.00 | Cervical disc disorder with, myelopathy, unspecified cervical region |
| 3 | 722.72 | Disc herniation w/myelopathy | M51.04 | Intervertebral disc disorders with myelopathy, thoracic region |
| 3 | 722.72 | Disc herniation w/myelopathy | M51.05 | Intervertebral disc disorders with myelopathy, thoracolumbar region |
| 3 | 722.73 | Disc herniation w/myelopathy | M51.06 | Intervertebral disc disorders with myelopathy, lumbar region |
| 3 | 722.73 | Disc herniation w/myelopathy | M51.07 | Intervertebral disc disorders with myelopathy, lumbosacral region |
| 3 | 722.81 | Post laminectomy syndrome | M96.1 | Postlaminectomy syndrome, not elsewhere classified |
| 3 | 722.83 | Post laminectomy syndrome | M96.1 | Postlaminectomy syndrome, not elsewhere classified |
| 3 | 722.83 | Post laminectomy syndrome | M96.1 | Postlaminectomy syndrome, not elsewhere classified |
| 3 | 722.91 | Disc space infection | M50.80 | Other cervical disc disorders, unspecified cervical region |
| 3 | 722.91 | Disc space infection | M50.90 | Cervical disc disorder, unspecified, unspecified cervical region |
| 3 | 722.92 | Disc space infection | M46.45 | Discitis, unspecified, thoracolumbar region |
| 3 | 722.92 | Disc space infection | M51.84 | Other intervertebral disc disorders, thoracic region |
| 3 | 722.92 | Disc space infection | M51.85 | Other intervertebral disc disorders, thoracolumbar region |
| 3 | 722.93 | Disc space infection | M51.86 | Other intervertebral disc disorders, lumbar region |
| 3 | 722.93 | Disc space infection | M51.87 | Other intervertebral disc disorders, lumbar region |
